# Comparison of DNA vaccines with AddaS03 as an adjuvant and an mRNA vaccine against SARS-CoV-2

**DOI:** 10.1016/j.isci.2023.107120

**Published:** 2023-06-16

**Authors:** Praveen Neeli, Dafei Chai, Xu Wang, Navid Sobhani, George Udeani, Yong Li

**Affiliations:** 1 Department of Medicine, Baylor College of Medicine, Houston, TX 77030, USA; 2 Department of Pharmacy Practice, Irma Lerma Rangel School of Pharmacy, Texas A&M University, Kingsville, TX 78363, USA

**Keywords:** Virology

## Abstract

Emerging variants of SARS-CoV-2 call for frequent changes in vaccine antigens. Nucleic acid-based vaccination strategies are superior as the coding sequences can be easily altered with little impact on downstream production. mRNA vaccines, including variant-specific boosters, are approved for SARS-CoV-2. Here, we tested the efficacy of DNA vaccines against the SARS-CoV-2 Spike aided by the AddaS03 adjuvant using electroporation and compared their immunogenicity with an approved mRNA vaccine (mRNA-1273). DNA vaccination elicited robust humoral and cellular immune responses in C57BL/6 mice with Spike-specific antibody neutralization and T cells produced from 20 μg DNA vaccines similar to that from 0.5 μg mRNA-1273. Furthermore, a Nanoplasmid-based vector further increased the immunogenicity*.* Our results indicate that adjuvants are critical to the efficacy of DNA vaccines in stimulating robust immune responses against Spike, highlighting the feasibility of plasmid DNA as a rapid nucleic acid-based vaccine approach against SARS-CoV-2 and other emerging infectious diseases.

## Highlights

• Spike DNA vaccines with AddaS03 (AD03) elicit potent neutralizing antibodies in mice
• Spike DNA vaccines produce a robust T cell response comparable to mRNA-1273 in mice
• Reducing vector size increases antigen expression and immune responses in mice
• DNA plus AD03 is comparable to mRNA-1273 at a proportional dose used in humans

## Graphical abstract

**Figure:**
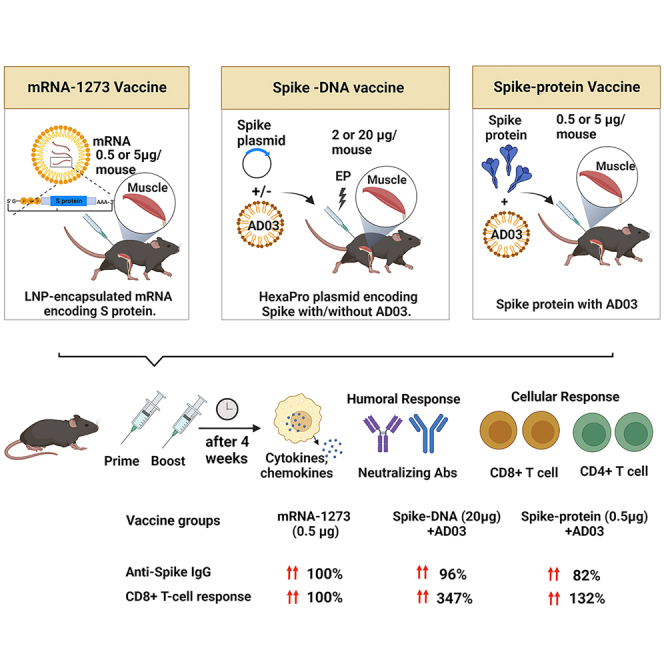

## Introduction

COVID-19 has imposed unprecedented morbidity and mortality for more than two years and continues to devastate global health and economies. More than 760 million people have been infected with SARS-CoV-2, and nearly 7 million people have died globally as of May 2023 since the outbreak of COVID-19 began. Vaccines effectively control the pandemic and help restore the global economy.^1^^,^^2^ Pharmaceutical and academic researchers have developed vaccines based on the inactivated virus, viral proteins, adenoviral vectors, and RNA or DNA for SARS-CoV-2. Nucleic acid-based vaccines like mRNA vaccines have been recognized as next-generation vaccines. However, the ultralow-temperature storage of mRNA limits its global availability. In contrast, DNA is very stable even at room temperature. Clinical trials of over a dozen of DNA-based COVID-19 vaccines are currently registered, and most use the electroporation (EP) method for delivery. EP enhances the entry of DNA plasmids into cells, resulting in increased expression in the skeletal muscle up to 100 times.^3^^,^^4^^,^^5^^,^^6^^,^^7^^,^^8^ DNA delivery via EP also activates immune cells to secrete cytokines to enhance immune response, as electric pulses may induce inflammatory responses and facilitate DNA vaccine efficacy *in vivo*.^9^

This work aimed to develop DNA vaccines against SARS-CoV-2 using DNA plasmids expressing the Spike protein. The DNA vaccines were aided by the AddaS03 from Invivogen (AD03), a squalene-based immunologic adjuvant. We compared the DNA vaccines with mRNA-1273 (Spikevax) from Moderna and the Spike protein. In addition, we introduced Nanoplasmids to reduce vector size for increasing antigen expression and improving immune responses. Together, our data suggest that the Nanoplasmid-based DNA vaccine with AD03 is a promising agent against SARS-CoV-2.

## Results

### Spike-DNA vaccine expression and validation

**Figure 1:**
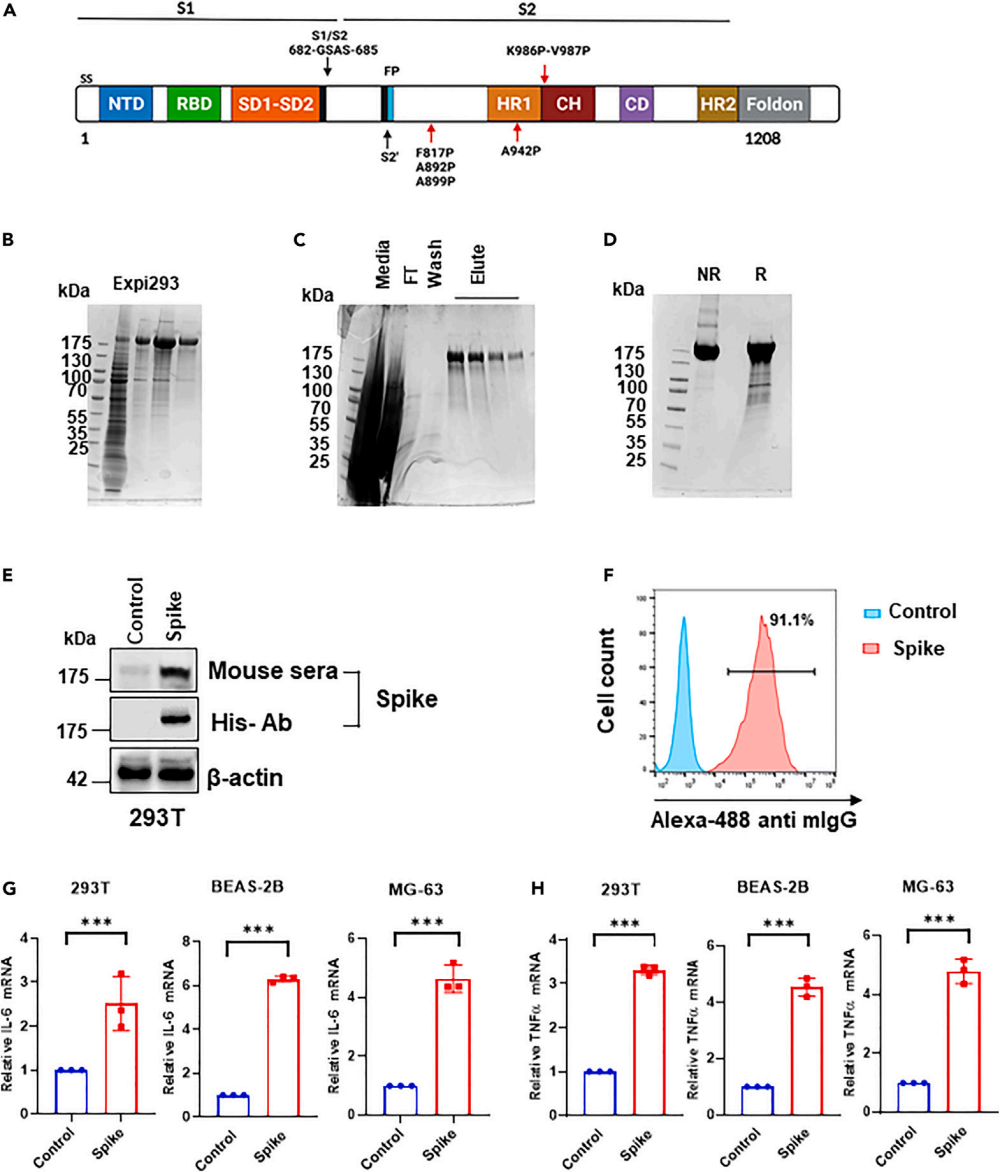
The recombinant Spike Protein as a DNA-vaccine candidate for SARS-CoV-2 (A) Schematic representation of the prefusion-stabilized SARS-CoV-2 HexaPro ectodomain showing the S1 and S2 subunits. Four additional proline substitutions from the S-2P construct are indicated by the red arrows shown below the construct. SS- Signal sequence; NTD N-terminal domain; RBD- Receptor Binding domain; SD1-2- Subdomain1-2; CH- Central helix; CD-connector domain; HR-heptad repeat FP- fusion peptide. (B) HexaPro Spike protein expressed in Expi293 cells was confirmed by SDS-PAGE. (C–D) His-tagged HexaPro was expressed in Expi293, purified and characterized by SDS-PAGE (right). (E) Expression of HexaPro Spike confirmed by western blot using a commercial anti-His antibody or pooled sera of Spike-immunized mice. (F) Flow cytometric analysis showing the binding of pooled mouse sera of HexaPro immunized mice to the HexaPro Spike expressed on 293T cells. (G–H) The mRNA expression levels of inflammatory cytokines IL-6 and TNFα in HexaPro-transfected cells were detected by qRT-PCR. The bars represent the means with error bars denoting the SD of three samples (∗∗∗significantly different (p < 0.001) by two-tailed unpaired t-test).

The prefusion-stabilized HexaPro Spike, constructed by Dr. McLellan, consists of the ectodomain of SARS-CoV-2 Spike with six-proline substitutions at residues 817, 892, 899, 942, 986, and 987, “GSAS” substitution at residues 682–685 (to remove the furin cleavage site), and C-terminal fold-on trimerization motif (Figure 1A).^10^ Transient transfection of the Spike plasmid based on the pαH vector^10^ into Expi293 cells resulted in protein expression in the culture supernatant (Figure 1B). The twin-Strep-tagged Spike proteins were purified using Strep-Tactin XT 4 flow kit and concentrated using Amicon centrifugal spin columns (Figure 1C). We confirmed the monomeric state of the purified Spike protein by SDS-PAGE (Figure 1D) and validated its expression *in vitro* using 293T cells by western blot (Figure 1E). Similarly, pooled sera of C57BL/6 mice immunized with 2-doses of Spike-DNA vaccines (20 μg per mice at two week-intervals; n = 5 animal group) show enhanced binding to Spike displayed on the cell surface of 293T (Figures 1E and 1F). This suggests that some Spike proteins are anchored onto the plasma membrane even without the transmembrane (TM).^10^ Next, qRT-PCR data show that mRNA expression levels of inflammatory cytokines IL6 and TNF-α were significantly elevated by Spike-plasmid in all the transfected cell lines compared with control (Figures 1G and 1H).

### Spike-DNA vaccination leads to enhanced humoral immune response and neutralizing activity against SARS-CoV-2 in the sera of vaccinated mice

**Figure 2:**
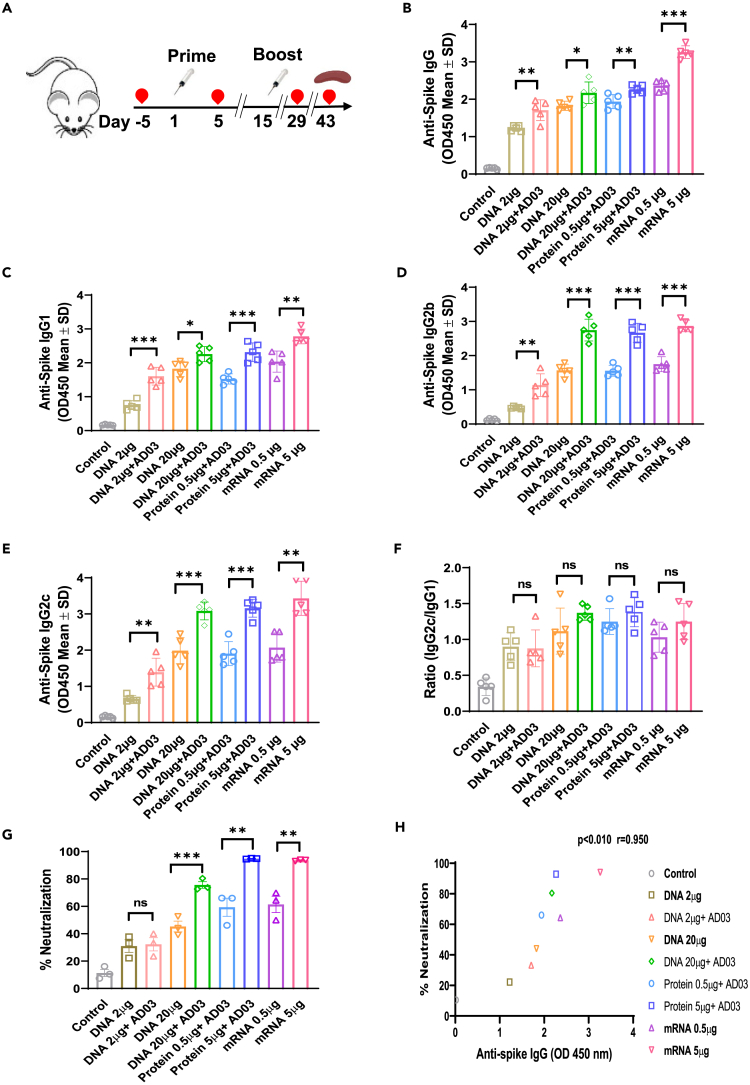
Spike-DNA vaccination induces a potent humoral response in immunized mice (A) Scheme of the immunization and immunological characterization. Four weeks after the second vaccination, blood was collected to detect IgGs. (B–E) Spike-specific mIgG (B), Anti-Spike mIgG1 (C), Anti-Spike mIgG2b (D), and Anti-Spike mIgG2c (E) levels measured using ELISA coated with HexaPro Spike protein. (F) The ratio of IgG2c/IgG1 was measured to gauge the Th1-type immune response in vaccinated mice. (G) Potent neutralizing Abs in the vaccinated sera against SARS-CoV-2 Spike protein was measured using the cPass neutralization kit. (H) Correlation between anti-Spike IgG and percentage of neutralization in immunized mice groups (*r* = 0.95, p < 0.01). The bars represent the means with error bars denoting the SD of five samples. Significant differences (ns = not significantly different; ∗p < 0.05; ∗∗p < 0.01; ∗∗∗p < 0.001; two-tailed unpaired t-test) are shown. Pearson’s correlations were calculated to define correlations.

We immunized C57BL/6 mice, following a schedule of prime-boost at 2-week intervals, with three types of vaccines. For the DNA groups, 2 μg (low dose) or 20 μg (high dose) of the Spike-plasmid^10^ or a control plasmid with or without AD03 were delivered IM followed by EP using the ICHOR electroporation system.^11^ The protein subunit group mice were IM vaccinated with either 0.5 μg or 5 μg of the purified HexaPro Spike protein with AD03. The mRNA groups received either 0.5 μg or 5 μg of mRNA-1273 IM (Figure 2A, Table 1). Antigen expression was confirmed by measuring the anti-Spike IgG levels in mice sera before immunization and 5 days after the first vaccination (Figure S1). Four weeks after the second vaccination, mice sera were collected to detect anti-Spike total IgG, IgG subtypes IgG1, IgG2b, and IgG2c by ELISA (Figures 2B–2E). Immunization with 2 μg DNA resulted in a significant increase in anti-Spike IgG than the control, but lower than that with 20 μg DNA. The addition of AD03 increased the levels in each IgG category for the DNA vaccines. In either low dose or higher dose, the mRNA vaccines exhibit the best or comparable IgG levels, compared to DNA or protein vaccines with AD03 (Figures 2B–2E). Consistent with the literature,^12^ all vaccinated mice showed a Th1-bias response based on the ratio of IgG2c and IgG1 (Figure 2F). We did not find a difference in the magnitude of IgG subtype responses between the vaccine groups. We noted a Th1-type bias in the 20 μg DNA with AD03 groups comparable to that of the 5 μg mRNA group (Figure 2F).

**Table 1:** Comparison of three Spike-targeting vaccine platforms in mice

Platform	DNA		mRNA		Protein	
Description	Plasmids encoding HexaPro Spike		mRNA (S-2P) in LNPs (mRNA-1273)		Purified HexaPro Spike	
Delivery	IM plus EP		IM		IM	
Doses	2 μg+AD03	20 μg+AD03	0.5 μg (LNPs)	5.0 μg (LNPs)	0.5 μg+AD03	5.0 μg+AD03
Anti-Spike IgG (%)	72	96	100	138	82	96
Neutralization (%)	52	123	100	152	96	145
IFNγ+CD4^+^ T cells (%)	134	181	100	220	80	159
IFNγ+CD8^+^ T cells (%)	153	347	100	400	132	290

To evaluate the anti-Spike antibodies binding to the Spike protein exogenously expressed on 293T cells, we tested the binding ability of serum antibodies of immunized mice by flow cytometry (Figure S2). Pooled sera of 2 μg or 20μg DNA-vaccinated mice allowed the detection of 25% or 40% of 293T cells that expressed Spike. Sera from AD03-adjuvanted DNA vaccination increased the numbers to 33% or 61.1%. The sera from mice immunized with 0.5 μg or 5 μg mRNA-1273 showed 29% or 96.6%, compared to 31% or 54.6% from the 0.5 μg or 5 μg protein group (Figure S2).

Next, we evaluated the functional activity of the antibodies using the cPass neutralization assay, which measures the reduction in the binding of Spike (the viral receptor binding protein) to the human ACE2 receptor by neutralizing antibodies (nAbs). The percentage of neutralization from anti-Spike sera (nAbs) of the 20 μg DNA group reached that from 0.5 μg mRNA, but less than that of the 5 μg mRNA or 5 μg protein group, which potentially had the highest levels of nAbs (Figure 2G). A significant positive correlation between anti-Spike total IgG and the percentage of neutralization was observed among various groups (Figure 2H; Pearson correlation coefficient *r* = 0.95, p < 0.010).

### DNA vaccination induced cellular responses against SARS-CoV-2 Spike in splenocytes of vaccinated mice

**Figure 3:**
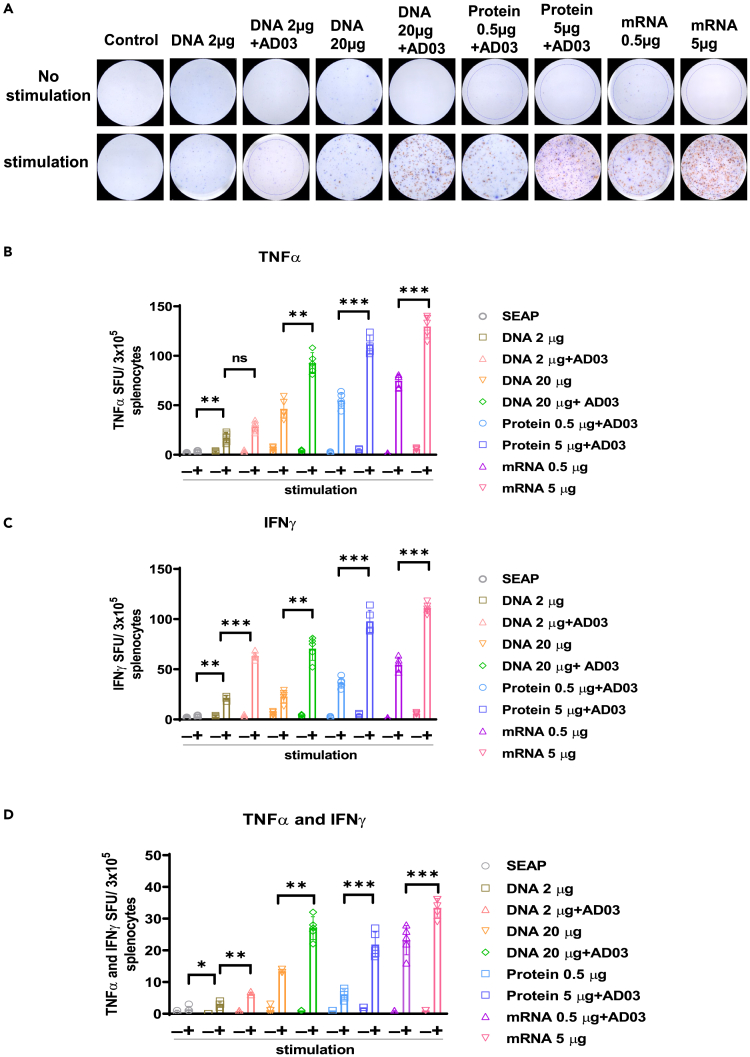
Spike-DNA vaccine potentiates functional T cells specific to SARS-CoV-2 Spike protein (A) C57BL/6 mice (n = 5/group) were immunized with indicated vaccine groups. Lymphocytes in the spleens were collected from each group after four weeks of second immunization and stimulated with overlapping peptide pools spanning full-length Spike protein for 24 h and measured stimulated splenocytes using a TNFα and IFNγ double color ELISpot assay kit. Blue spots represent TNFα, and red spots represent IFNγ. (B) Count of TNFα producing T cells. (C) Count of IFNγ producing T cells. (D) Count of TNFα and IFNγ double-positive T cells. The bars represent the means with error bars denoting the SD of five samples. Significant differences (ns = not significantly different; ∗p < 0.05; ∗∗p < 0.01; ∗∗∗p < 0.001; one-way ANOVA) among different groups are shown in the corresponding figures.

**Figure 4:**
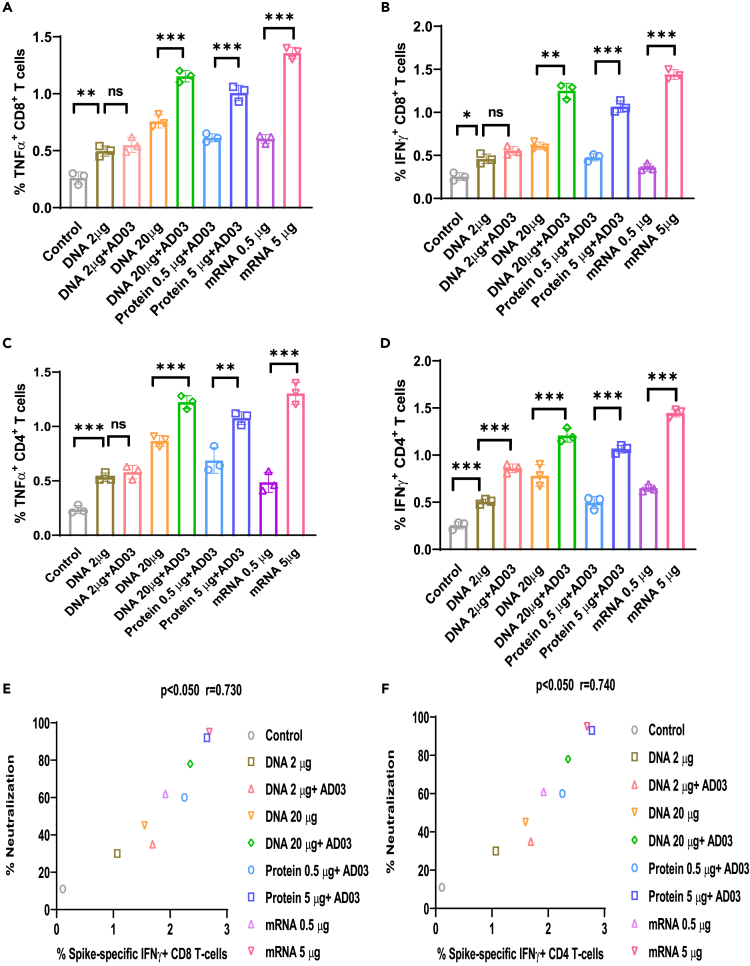
Spike-DNA vaccine induces CD8^+^ and CD4^+^ T cell responses specific to SARS-CoV-2 Spike protein Splenocytes (3x10^6^/mL) from indicated vaccinated mice groups were stimulated with SARS-CoV-2 peptide pool (5 μ*g*/*mL*). (A–D) (A) TNFα+ CD8^+^ T-cells, (B) IFNγ+ CD8^+^ T-cells, (C) TNFα+ CD4^+^ T-cells, (D) IFNγ+ CD4^+^ T-cells population was analyzed using Flow cytometry. (E) Correlation between Spike-specific IFNγ+ CD8^+^ T-cells and percentage of neutralization in immunized mice groups (p < 0.05, r = 0.73). (F) Correlation between Spike-specific IFNγ+ CD4^+^ T-cells and percentage of neutralization in vaccinated mice groups (p < 0.05 r = 0.74). Significance differences (ns = not significantly different; ∗p < 0.05; ∗∗p < 0.01; ∗∗∗p < 0.001; two-tailed unpaired t-test) are shown and the data are presented as the means ± SD of at least three samples. Pearson’s correlations were calculated to define correlations.

We next evaluated the induction of systemic cytokines and cellular responses in mice at 4 weeks after the second immunization using TNFα and IFNγ double ELISpot assay and flow cytometric analysis of splenocytes. Isolated splenocytes were stimulated with 5 μg/mL of the Spike peptide pool for 24 h. ELISpot (Figures 3A–3D) showed that splenocytes from mice immunized with 20 μg DNA with AD03, 5 μg mRNA1273, or 5 μg protein led to the highest induction of TNFα- and IFNγ-producing splenocytes (Figures 3A–3D). In line with ELISpot, flow cytometry revealed that the percentages of TNFα- and IFNγ-positive cytotoxic CD8^+^ T cells followed the rank of 5 μg mRNA, 20μg DNA+AD03, 5 μg protein, 20μg DNA, 0.5 μg mRNA, 0.5 μg protein, 2 μg DNA+AD03, and 2μg DNA (Figures 4A, 4B, and S4). Similar ranking among the vaccinated groups was observed for IFNγ- and TNFα-producing CD4^+^ T cells (Figures 4C and 4D). There was a positive correlation between the percentages of neutralization and IFNγ-producing CD4 and CD8 T cell populations among various vaccinated groups (nAb: IFNγ+ CD8 T cells, p < 0.05, r = 0.73) (nAb: IFNγ+ CD4 T cells, p < 0.05 r = 0.74; Figures 4E and 4F). Notably, 20 μg DNA with AD03 led to more IFNγ-positive CD4^+^ or CD8^+^ T cells than 0.5 μg mRNA but fewer than 5 μg mRNA.

**Figure 5:**
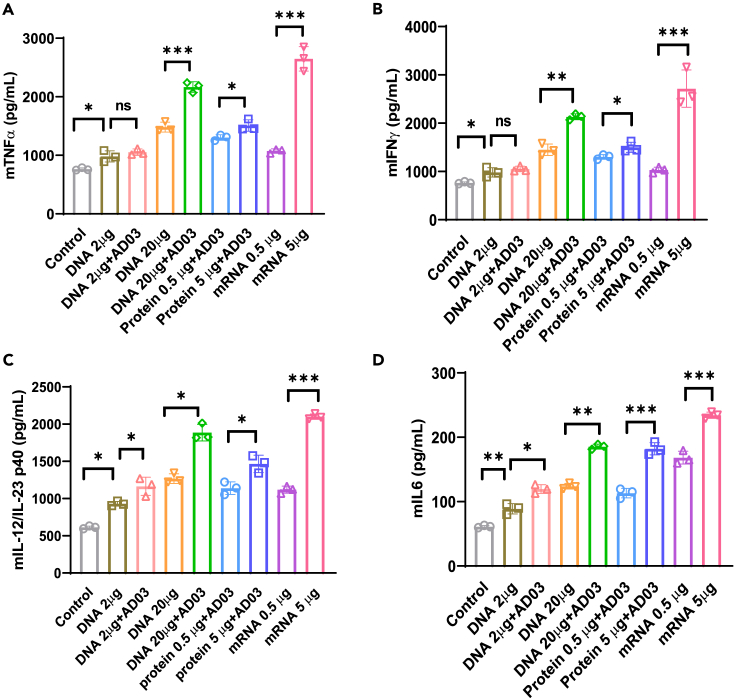
Spike-DNA vaccination induces proinflammatory cytokine production in Spike-stimulated splenocytes Splenocytes (3x10^6^/mL) from indicated vaccinated mice groups were stimulated with SARS-CoV-2 peptide pool (5 μ*g*/*mL*) for three days, and secretion of cytokines. (A–D) (A) TNFα (B) IFNγ (C) IL-12p40 (D) IL-6 from splenocytes was detected by ELISA. Significant differences (ns = not significantly different; ∗p < 0.05; ∗∗p < 0.01; ∗∗∗p < 0.001; two-tailed unpaired t-test) are shown and the data are presented as means ± SD of at least three samples.

We next characterized the induction of systemic cytokines in response to the SARS-CoV-2 Spike antigen using ELISA. Splenocytes of vaccinated mice were stimulated with the Spike protein (5 μg/mL), and cytokine secretion in cell culture supernatants was detected 3 days post-stimulation. Three Th1 cytokines (TNFα, IL-12p40, and IFNγ) and one Th2 cytokine (IL-6) were measured. All were significantly increased in the 20 μg DNA with the AD03 group at a level comparable to 0.5 μg mRNA or 5 μg protein but lower than 5 μg mRNA (Figures 5A–5D). The level of IL-6 was an order of magnitude lower than those of Th1 cytokines. These data suggest that 20 μg DNA with AD03 led to an enhanced cellular immune response skewed to the Th1 response when exposed to the SARS-CoV-2 Spike antigen.

### Nanoplasmids expressing spike elicit a stronger humoral and cell-mediated immune response than the conventional plasmid in the vaccinated mice

**Figure 6:**
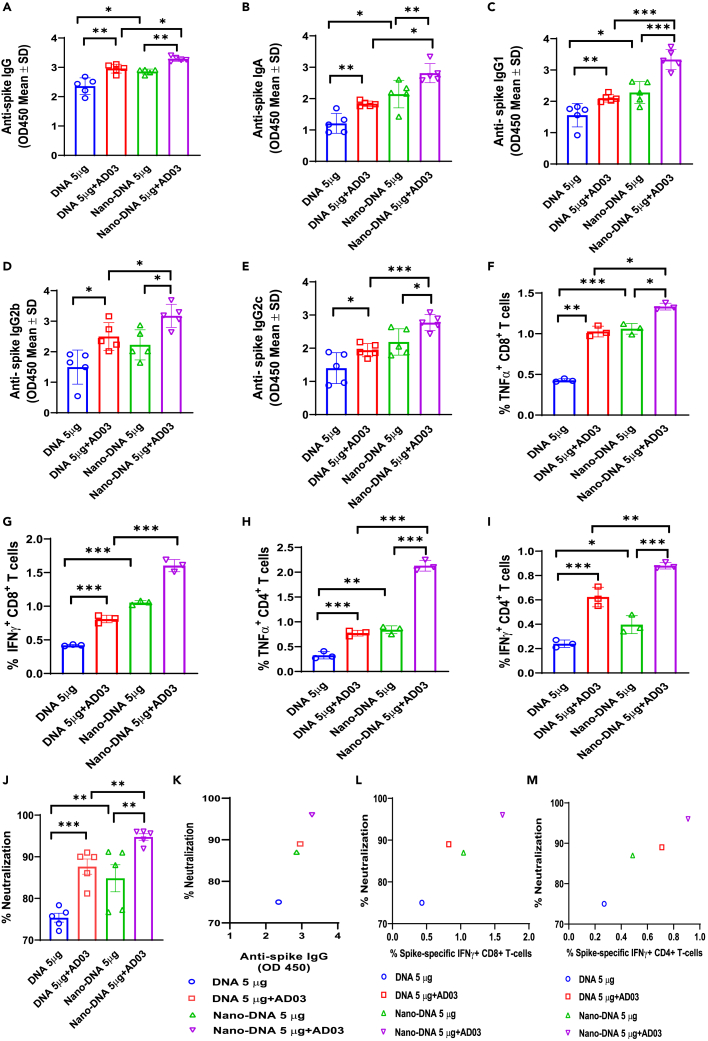
Mice immunized with Nano-DNA exhibit higher IgG and T cell responses than conventional plasmid; Adjuvant AD03 potentiates immune responses C57BL/6 mice were immunized with conventional Spike (Spike-DNA) or Nano-DNA plasmids (5μg/mice) with or without adjuvant-AD03 twice, at two weeks interval periods. Four weeks after the second vaccination, blood was collected to detect. (A–I) (A) Spike-specific mIgG (B) Anti-Spike mIgA (C) Anti-Spike mIgG1 (D) Anti-Spike mIgG2b (E) Anti-Spike mIgG2c levels using ELISA coated with Spike protein (5 μg/mL). Splenocytes collected at four weeks after the second vaccination (3x10^6^/mL) were stimulated with SARS-CoV-2 Spike peptide pool (5 μ*g*/*mL*) and measured the (F) TNFα+ CD8^+^ T-cells, (G) IFNγ+ CD8^+^ T-cells, (H) TNFα+ CD4^+^ T-cells, (I) IFNγ+ CD4^+^ T-cells population at 3 days post-stimulation using Flow cytometry. (J) Neutralizing antibodies in the vaccinated sera against SARS-CoV-2 Spike protein were measured using the cPass neutralization kit. (K) Correlation between anti-Spike IgGs and percentage of neutralization. (L) Correlation between Spike-specific IFNγ+ CD8^+^ T-cells and percentage of neutralization in immunized mice groups. (M) Correlation between Spike-specific IFNγ+ CD4^+^ T-cells and percentage of neutralization in immunized mice groups. Significant differences (ns = not significantly different; ∗p < 0.05; ∗∗p < 0.01; ∗∗∗p < 0.001; two-tailed unpaired t-test) are shown and the data are presented as means ± SD of at least three samples.

We further improved the Spike-DNA Vaccine using the Nanoplasmid technology and compared its efficacy with the conventional plasmid (DNA: pαH-based^10^) *in vitro* and *in vivo*. Nanoplasmids are small circular DNA constructs without antibiotic-resistance genes that can be produced in large yields. The open reading frame of HexaPro Spike was cloned into the Nanoplasmid NTC9385R-eRNA41H-CpGRNA vector (Nano-DNA; Figure S3). Expi293T cells transfected with Nano-DNA produced more Spike after purification than pαH-Spike (DNA) (Figure S5A). C57BL/6 mice immunized with 5 μg Nano-DNA had elevated Spike antigen levels in their sera compared to mice with 5 μg DNA (Figure S5B). Next, we immunized C57BL/6 mice twice, following a schedule of prime-boost at 2-week intervals with 5 μg of DNA or Nano-DNA with or without AD03 using EP. At 4 weeks post-second vaccination, the sera were collected from immunized mice to detect the anti-Spike IgG, IgA, and IgG subtypes using ELISA. Mice vaccinated with Nano-DNA showed higher levels of anti-Spike IgG, IgA, IgG1, IgG2b, and IgG2c than DNA (Figures 6A–6E). The AD03 adjuvant further increased the antibody levels (Figures 6A–6E). We then characterized the cellular response of systemic cytokines in response to vaccination. Flow cytometry revealed that the percentages of TNFα- and IFNγ-positive cytotoxic CD8^+^ T cells followed the rank of Nano-DNA+AD03, Nano-DNA, DNA+AS03, and DNA (Figures 6F and 6G). The percentages of IFNγ- and TNFα-positive CD4^+^ T cells were elevated in the Nano-DNA+AD03 group compared to Nano-DNA alone or DNA+AS03 groups (Figures 6H and 6I). Similar patterns were observed for the percentages of neutralization (Figures 6J and 6K). The highest neutralization was achieved by Nano-DNA+AD03. Again, the neutralization levels were positively correlated with the numbers of IFNγ-secreting CD8^+^ cells (Figure 6L) or CD4^+^ T cells (Figure 6M).

**Figure 7:**
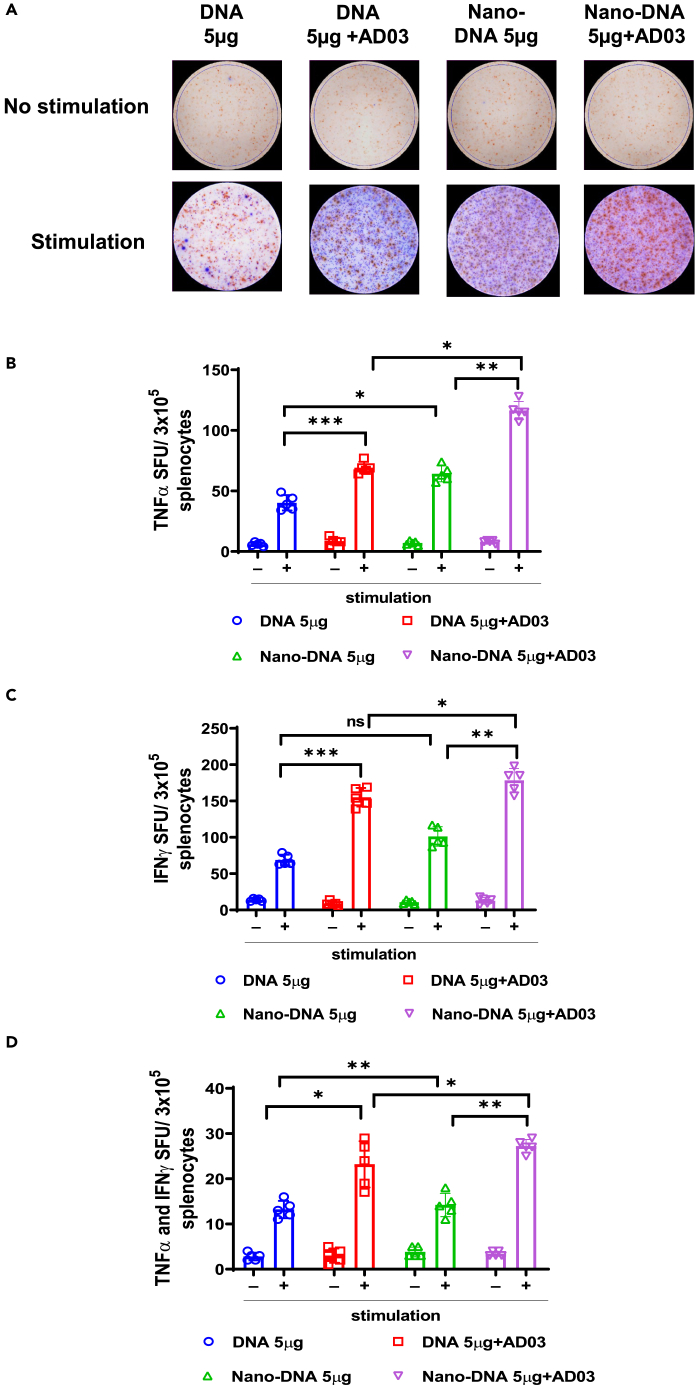
Mice immunized with Nano-DNA potentiate functional T cells specific to SARS-CoV-2 Spike compared to the conventional plasmid (A) C57BL/6 mice (n = 5/group) were immunized with indicated vaccine groups. Lymphocytes in the spleens were collected from each group after four weeks of second immunization, stimulated with SARS-CoV-2 Spike peptide pool for 24 h, and measured stimulated splenocytes using a TNFα and IFNγ double color ELISpot assay kit. Blue spots represent TNFα; Red spots represent IFNγ. (B–D) (B) Count of TNFα producing T cells (C) Count of IFNγ producing T cells (D) Count of TNFα and IFNγ double-positive T cells. The bars represent the means with error bars denoting the SD of five samples. Significant differences (ns = not significantly different; ∗p < 0.05; ∗∗p < 0.01; ∗∗∗p < 0.001; one-way ANOVA) are shown.

Next, we measured the induction of systemic cytokines TNFα and IFNγ using ELISpot. Splenocytes from mice immunized with Nano-DNA+AS03 produced more TNFα- or IFNγ-positive splenocytes than other groups (Figures 7A–7D). These data suggest that Nano-DNA with or without AD03 show enhanced cellular immune responses than their conventional counterparts.

## Discussion

The past few years have observed the unparalleled successes of mRNA-based COVID-19 vaccines against SARS-CoV-2. Yet there is still a disparity in vaccine equity, access, and supply to low- and middle-income countries, as over 2 billion people remain completely unvaccinated. The anti-Spike IgG and nAb levels were correlated with the percentage of protection of the vaccines,^13^ and the severity of COVID-19 inversely correlates with anti-Spike antibodies.^14^ The two mRNA vaccines produced about 4-fold nAbs over the convalescent plasma and showed over 94% efficacy, much higher than conventional vaccines based on the inactivated virus.^15^^,^^16^ However, mRNA vaccines need ultra-low temperatures for storage, a barrier for many low- or middle-income countries. DNA vaccines delivered via EP are thermally stable and easy to manufacture, yet their efficacies in protection are not on par with mRNA vaccines.^17^ The INO-4800 DNA vaccine from Inovio Inc. elicited favorable T cell responses, but the nAb levels were only ∼10% of the convalescent plasma.^18^ A needle-free intradermal COVID-19 DNA vaccine (ZyCov-D) developed by Cadila Healthcare Inc. had low humoral and cellular immune responses with a 1,000 μg dosage. In comparison, the 2,000 μg ZyCov-D dose group with three injections had moderate IgG, nAb, and T cell responses, with a protective efficacy of 66.6% in patients.^19^ Notably, the Spike antigens from these two DNA vaccines are not explicitly stated to contain the two proline (S-2P) substitutions critical for maintaining the Spike protein in a prefusion conformation.^20^^,^^21^ In this study, we used DNA plasmids expressing the newest prefusion stabilized Spike antigen (HexaPro, the 2P form plus four additional residues substituted by proline).^10^^,^^22^ HexaPro Spike is more stable than S-2P. Preclinical studies have proved that HexaPro is an ideal candidate for development of new vaccines.^23^^,^^24^ In human DNA vaccine clinical trials, 2,000 μg of DNA were frequently used, whereas 30 or 100 μg of mRNA were included in the two approved mRNA vaccines. Specific to mRNA-1273, doses ranging from 0.0025 to 20 μg per mouse were tested with 0.2 and 1.0 μg inducing robust pseudovirus-neutralizing activity and CD8 T+ cell responses.^25^ Thus, we considered 20 μg DNA in mice are proportional to 2,000 μg in humans, comparable to 0.5 μg of mRNA in mice when scaled to 50 μg in humans. There are over 20 studies testing DNA vaccines against Spike of SARS-CoV-2 (Table S1), yet none has compared DNA with mRNA. This is the first study to simultaneously compare the immunogenicity of DNA, mRNA, and protein vaccines in mice. We demonstrate that 20 μg of DNA plus AD03 achieve IgG and neutralization at levels similar to 0.5μg of mRNA (Figure 2, Table 1).

Adjuvants are critical for improving the quality and magnitude of immune responses.^26^ There are no adjuvants in INO-4800 or ZyCov-D. We noticed that the Inovio group added a CC- chemokine receptor 10 (CCR10) as an adjuvant in their upgraded synthetic DNA vaccine against SARS-CoV-2.^27^ The mRNA vaccines are mainly composed of cationic lipids like SM-102, stabilizer lipids like PEG2000-DMG, helper lipids (distearoylphosphatidylcholine), and cholesterol, each serving distinct functions.^28^ Ionizable lipid SM102 facilitates the intracellular delivery of LNPs, while other helper lipids stabilize the LNP.^29^ Protein antigens delivered with LNPs (without encapsulation) elicited strong T helper cell and humoral responses, indicating that LNPs within mRNA vaccines not only facilitate delivery but also possess the strong adjuvant activity and enhance the immunogenicity of protein subunit vaccines.^26^ These data support that sufficient adjuvants are critical to the success of nucleic acid-based vaccination. AD03 is an oil-in-water emulsion composed of squalene, polysorbate 80, and α-tocopherol, which potently induces antibodies and increases vaccine durability, promoting heterologous cross-reactivity, and having antigen dose-sparing effect (fewer antigens used for vaccination).^30^ In a study comparing different adjuvants with the same amounts of Spike antigens, AD03 exhibits superior efficacy to other classical adjuvants like AS37, CpG1018, and alum in protecting experimental animals from SARS-CoV-2 infection.^31^^,^^32^ The addition of AD03 in our vaccination increased the neutralization levels significantly in the 5 μg and 20 μg DNA groups, supporting that adjuvants augment the efficacy of DNA vaccines delivered via EP.

Further, we improved the efficacy of the Spike-DNA vaccine through vector engineering. The ZyCoV-D used the pVAX1 as the vector,^19^ and INO-4800 used pGX9500, a modified version of pVAX1.^18^^,^^33^ Next-generation vector designs improve antigen expression, manufacturing yield and quality, and regulatory compliance.^34^ The Nanoplasmid NTC9385R-eRNA41H-CpGRNA vector was developed by Nature Technology Corporation (Lincoln, NE) for DNA vaccination. *First*, Nanoplasmids bypass the antibiotic selection when amplified in *E. coli*. They incorporate and express a 150 bp RNA-OUT antisense RNA. RNA-OUT represses the expression of a chromosomal counter-selectable marker (SacB),^35^ which encodes a levansucrase, a toxin to *E. coli*, in the presence of sucrose. Plasmid selection is achieved in sucrose-containing media. *Second*, Nanoplasmids contain highly productive heat-inducible R6K origins for DNA replication. Under a fermentation process called HyperGRO,^36^^,^^37^ DNA yields of up to 2,400 mg/L have been obtained with Nanoplasmid vectors. *Third*, Nanoplasmids have an optimized chimeric promoter-intron (CMV-HTLV-I R synthetic intron), thereby achieving significantly higher expression levels (2- to 10-fold) than pVAX1, which has no intron but has the same CMV promoter and enhancer as Nanoplasmids (and bovine growth hormone polyadenylation signal).^34^^,^^35^ Nanoplasmids use a backbone <0.5 kb (∼2.0 kb for pVAX1) for plasmid preparation (replication and selection). *Finally*, the NTC9385R-eRNA41H-CpGRNA plasmid contains two adjuvant elements. (1) eRNA41H is a 114 bp RNA fragment engineered to induce type I interferon (IFN) production through the activation of retinoic acid-inducible gene 1 (RIG-I).^38^ (2) CpG RNA is a potential agonist of alternative innate immune receptors that activate adaptive immunity.^39^^,^^40^ We use the Nanoplasmid NTC9385R-eRNA41H vector to express Spike HexaPro for maximized antigen expression and immunogenicity. The final construct (Nano-DNA; 6,452 bp; with the CMV promoter and the CMV-HTLV-I R synthetic intron) is smaller than the pαH-based parental vector (DNA; 8,370 bp, with the chicken β-actin promoter and a chimeric intron from chicken β-actin and rabbit β-globin)^10^; both plasmids use the same CMV enhancer and the rabbit β-globin polyadenylation signal. Nano-DNA elicits a more robust immune response than the parental vector, which is further enhanced by the addition of AD03. Five μg of Nano-DNA exhibit an IgG level and a percentage of neutralization comparable to 5.0 μg mRNA, *albeit* the assays are not performed in parallel. We noted that a previous study used an earlier version of Nanoplasmid to target Spike.^41^ Yet it required 3 doses of 10–50 μg plasmid DNA. Nonetheless, our data support that adding adjuvants like AD03 to the 9th iteration of Nanoplasmids, which already express eRNA41H and CpG RNA as adjuvants, is needed to maximize the immune responses from DNA vaccination.

Vaccines that generate potent nAbs and Th1-biased T cell responses reduce the risk of antibody-dependent replication enhancement. INO-4800 T cell response was close to convalescent plasma (albeit with a small number of patients).^18^^,^^42^ ZyCov-D induced minimal cellular response compared with the placebo group.^43^ In our Spike-DNA vaccines, the T cell response from 20 μg DNA plus AD03 was higher than that of 0.5 μg mRNA and close to that from 5.0 μg mRNA (Table 1). Nanoplasmid DNA vaccines further improved cellular responses. T cell response plays a central role in inducing anti-tumor response.^44^ In this context, DNA vaccination represents a promising strategy for eliciting adaptive immune responses to cancer. Easy delivery of multiple antigens and induction of cellular and humoral immunity without being restricted to HLA-patient type makes the DNA vaccines a promising cancer prevention and treatment strategy.

To summarize, the Spike-DNA vaccines aided by AD03 at 5–20 μg per mouse activate robust humoral and cellular responses specific to the SARS-CoV-2 Spike protein, comparable to 0.5 μg per mouse of a leading mRNA vaccine. Nanoplasmids express higher antigens than conventional plasmids, producing more robust immune responses. Together, this study underscores the importance of antigen design (i.e., 2P and HexaPro mutations), vector engineering, and adjuvant stimulation in DNA vaccines.

### Limitations of the study

There are several limitations to this study. First, because of technical restrictions, we could not reliably track and quantify the exact amount of plasmid DNA uptake in mouse tissues, which may be a critical factor that impacts the expression of Spike *in vivo*. Second, there are several differences between the mRNA-1273 and DNA (and Nano-DNA): (1) S-2P in mRNA and HexaPro Spike in DNA; (2) the C-terminal sequence (the TM domain plus the short intracellular domain in mRNA was replaced by a trimer foldon domain from T4 bacteriophage fibritin). The HexaPro Spike is more stable than S-2P, whereas the TM domain in S-2P allows Spike to anchor better to the plasma membrane and evoke more potent neutralizing antibodies. Third, there is no live virus to challenge the animals, which could help delineate how the enhanced humoral and cell responses may impact the pathogenicity of SARS-CoV-2. Lastly, our results were generated using mouse models, which may not completely mimic the characteristics of human immune responses.

## STAR★Methods

### Key resources table

**Table:** 

REAGENT or RESOURCE	SOURCE	IDENTIFIER
**Antibodies**		

Mouse Monoclonal anti-β-Actin antibody	Sigma-Aldrich	Cat#A2228
Rabbit Polyclonal anti-His antibody	Millipore Sigma	Cat#SAB1306084
Anti-rabbit IgG HRP-linked antibody	Cell Signaling Technology	Cat#7074
Anti-mouse IgG HRP-linked antibody	Cell Signaling Technology	Cat#7076
Goat Anti-Mouse IgG1 HRP-linked antibody	Abcam	Cat#97240
Goat Anti-Mouse IgG2c HRP linked antibody	Cell Signaling Technology	Cat#56970
Goat Anti-Mouse IgG2b HRP-linked antibody	Abcam	Cat#97250
Goat Anti-Mouse IgA HRP-linked antibody	Invitrogen	Cat#626720
APC anti-mouse CD4 antibody	Biolegend	Cat#100516; RRID:AB_312719
PerCP/Cyanine 5.5 anti-mouse CD8a antibody	Biolegend	Cat#100734; RRID:AB_2075238
Alexa Flour 488anti-mouse TNF-α antibody	Biolegend	Cat#506313; RRID:AB_493328
PE anti-mouse IFN-γ antibody	Biolegend	Cat#505808; RRID:AB_315402

**Bacterial and virus strains**		

DH5alpha chemically competent cells	New England Biolabs Inc.	Cat#C2987H

**Biological samples**		

Mouse serum (Immunized)	Stored in lab	N/A

**Chemicals, peptides, and recombinant proteins**		

AddaS03 (oil-in-water nano-emulsion aduvant)	Invivogen	Cat#vac-as03-10
SARS-CoV-2 Spike peptide Pool	Stemcell	Cat#100-0676
Cell stimulation cocktail 500X	Thermofisher Scientific	Cat#00-4970-93
Brefeldin A solution 1000X	Biolegend	Cat#420601
TRIzol reagent	Invitrogen	Cat#15596026
GlutaMAX	Thermo Fisher	Cat#35050061
Sodium pyruvate 100mM	Thermo Fisher	Cat#11360070
MEM Non-Essential Amino Acids solution	Thermo Fisher	Cat#11140050
L-Glutamine 200mM	Thermo Fisher	Cat#25030081
Trypsin-EDTA	Thermo Fisher	Cat#25030054
TMB Substate	Sigma-Aldrich	Cat#T0440
Dulbecco’s Modified Eagle Medium	Thermo Fisher Scientific	Cat#41966052
Expi293 Expression Medium	Thermo Fisher Scientific	Cat#A1435102
Fetal Bovine Serum	Thermo Fisher Scientific	Cat#10270106

**Critical commercial assays**		

cPass SARS-CoV-2 Neutralization Antibody Detection Kit	Genscript	Cat#L008475
Mouse IFN-γ/TNF-α Double-Color Elispot	Immunospot	Cat# mIFNgTNFa-1M
SARS-CoV2 Spike S1 AlphaLISA kit	PerkinElmer	Cat#AL3142C
iScript cDNA synthesis kit	Biorad	Cat#1708896
Expifectamine 293 transfection kit	Thermo scientific	Cat#A14525
Strep-Tactin purification Kit	Fisher Scientific-IBA Lifesciences	Cat# 2-5033-001
ZymoPURE II Plasmid Midiprep	Zymo Research	Cat#D4200
Mouse TNF-α Duoset ELISA	R&D systems	Cat#DY410-05
Mouse IFN-γ Duoset ELISA	R&D systems	Cat#DY485
Mouse IL-6 Duoset ELISA	R&D systems	Cat#DY406
Mouse IL-12/IL-23 p40 DuoSet ELISA	R&D systems	Cat#DY499

**Experimental models: Cell lines**		

Expi293F	Thermo Fisher	Cat#A14528
HEK-293T	ATCC	Cat#CRL3216
BEAS-2B	ATCC	Cat#CRL9482
MG-63	Tissue culture Core laboratory, Baylor college of Medicine	Cat#CRL1427 (ATCC)

**Experimental models: Organisms/strains**		

Mouse: C57BL/6; female	Jackson Laboratory	Strain #**000664**

**Oligonucleotides**		

IL6 Forward 5′-ACAGCCACTCACCTCTTCAG-3′	Sigma -Aldrich	N/A
IL6 Reverse 5′-CCATCTTTTTCAGCCATCTTT-3′	Sigma -Aldrich	N/A
TNF-α Forward 5′-CCCGAGTGACAAGCCTGTAG-3′	Sigma -Aldrich	N/A
TNF-α Reverse 5′-GATGGCAGAGAGGAGGTTGAC-3′	Sigma -Aldrich	N/A
GAPDH Forward 5′-GGATTTGGTCGTATTGGG-3′	Sigma -Aldrich	N/A
GAPDH Reverse 5′-GGAAGATGGTGATGGGATT-3′	Sigma -Aldrich	N/A

**Recombinant DNA**		

SARS-CoV-2 S HexaPro Plasmid	Addgene	Cat#154754
Nano-DNA NTC9385R-SARS-CoV-2 plasmid	Nature Technologies	N/A

**Software and algorithms**		

ImageJ	National Institutes of Health	https://imagej.nih.gov/ij/
GraphPad Prism version 8	GraphPad Software	https://www.graphpad.com/scientificsoftware/prism/
FlowJo software version 10.8.1	TreeStar Inc	https://www.flowjo.com/solutions/flowjo/downloads
Biorender	Biorender Software	https://app.biorender.com/
Snapgene Viewer Version 6.1.2	Snapgene Software	www.snapgene.com

### Resource availability

#### Lead contact

Further information and requests for resources and reagents should be directed to and will be fulfilled by the lead contact, Yong Li (Yong.Li@bcm.edu).

#### Materials availability

This study did not generate new unique reagents.

### Experimental model and subject details

#### Cell lines

BEAS2B, 293T cells were obtained from American Type Culture Collection (Manassas, VA). MG-63 was obtained from the Tissue Culture Core Laboratory, Baylor College of Medicine.

#### Plasmids

HexaPro Spike plasmid (DNA) was obtained from Addgene (#154754). Nano-DNA plasmid was generated by Nature Technology by subcloning the HexaPro-Spike insert into NTC9385R-eRNA41H-CpG RNA.

#### Mouse strains

C57BL/6 (#000664) mice were obtained from Jackson Laboratory (Bar Harbor, United States). All mice used for experiments were aged between 8-12 weeks, unless indicated otherwise.

### Methods details

#### Plasmid constructs

The mammalian expression plasmid encoding the SARS-CoV-2 HexaPro Spike with two Strep-Tag II and a His-Tag was obtained from Addgene (#154754). The reliability of the insert was confirmed by DNA sequencing. Subsequently, the recombinant plasmids were transformed into DH5a Escherichia coli competent cells and cultured in LB medium containing 100 mg/mL ampicillin overnight. The plasmids were isolated and purified from the bacteria by *Endo*-*Free* Plasmid DNA *Maxi Kit* and were identified by gel electrophoresis again. Nano-DNA plasmid was generated by Nature technology by subcloning the HexaPro Spike to NTC9385R-eRNA41H-CpG RNA using KpnI-transgene-XhoI. All plasmids were validated by sequencing.

#### Animals and cell lines

Six to eight-week-old C57BL/6 mice were purchased from Jackson Laboratory (Bar Harbor, ME) and maintained at Baylor College of Medicine Animal Facility. All procedures were carried out with IACUC approval at Baylor College of Medicine. Human kidney cell line 293T and human lung epithelial cell line BEAS2B cells were purchased from the American Type Culture Collection (Manassas, VA). MG-63 was obtained from Tissue Culture Core Laboratory, Baylor College of Medicine. 293T and BEAS2B and MG-63 were grown in complete Dulbecco’s modified Eagle medium (DMEM; Life Technologies, USA) containing 10% FBS and 100 U/ml of penicillin/streptomycin in a humid environment containing CO_2_ and air at 37°C. The human Expi293F cells were grown in Expi293 Expression Medium (Thermo scientific #A1435101). Splenocytes isolated from vaccinated mice were incubated in complete RPMI 1640 supplemented with murine IL-2 for six days with or without stimulation with the Spike peptide pool (5μg/ml).

#### Western blot analysis

Cells were lysed in RIPA lysis buffer (Thermo Fisher Scientific, Waltham, MA) containing a protease inhibitor cocktail and phosphatase inhibitors (Thermo Fisher Scientific) and then collected and centrifuged at 12,000 rpm for 15 min at 4°C. The supernatant was measured with the BCA protein assay reagent (Thermo Fisher Scientific). Lysates were denatured in Laemmli sample buffer (Bio-Rad, Hercules, CA) and resolved by Tris-glycine SDS-PAGE (4–20% polyacrylamide, Mini-PROTEAN Precast Gels, Bio-Rad). After transferring to the polyvinyl difluoride membrane, the membrane was blocked with 5% non-fat dry milk (Biorad #1706404) in 0.1% TBS-Tween-20 for 2 h and incubated with the primary antibodies at 4°C overnight. HRP-conjugated anti-rabbit or anti-mouse IgG (Cell Signaling Technology) was used as the secondary antibody. Immunoreactive protein was visualized with the enhanced chemiluminescent (ECL) western blotting substrate (Thermo Fisher Scientific #34076). Antibodies against β-actin (cat. #A5316) and His-tag (SAB1306084) were obtained from Sigma-Aldrich.

#### RNA extraction and qRT-PCR

Total RNA was extracted from TRIzol reagent (Invitrogen # 15596026) and reverse-transcribed into cDNA with iScript™ cDNA synthesis kit according to the manufacturer’s instructions (Bio-Rad #1708896). Gene expression levels were quantified by qRT-PCR performed on a QuantStudio 7 Pro qRT-PCR system (Thermofisher #A43183). The qRT-PCR was performed using primers for each gene, and the results were normalized to GAPDH transcript levels. The difference in fold expression was measured using the ΔΔCT method. Primers used against each gene were validated for specificity using BLAST and melting curve analysis.

#### Purification of recombinant SARS-CoV-2 HexaPro Spike Protein

Plasmids were transiently transfected into Expi293F using Expifectamine 293 transfection kit (Thermo scientific #A14525). Proteins were purified after six days of culture post-transfection. Cells and culture medium were separated by centrifugation at 3500 g for five minutes. The supernatant was concentrated with Amicon Ultra centrifugal filter unit (Millipore #UFC901024). Filtered supernatant containing the secreted protein was purified with Strep-Tactin resin as per the manufacturer instructions (IBA Biosciences, Göttingen, Germany). Elution fractions containing HexaPro were pooled and exchanged to phosphate-buffered saline (PBS) and concentrated using Amicon centrifugal spin columns. The purity of Spike proteins was measured by SDS-PAGE and western blot against His-tag antibody (#2365) or pooled immunized mice sera. The purified proteins were kept at −80°C in single-use aliquots. Each aliquot was thawed and briefly incubated (∼20 min) at 37 °C before use.

#### Mouse immunization

Total DNA (2 μg or 20 μg HexaPro plasmid or 20μg empty vector) in 10μL water was mixed with 10μL of AD03 adjuvant and injected into the anterior tibialis muscle (TA), followed by applying electrical fields with amplitude of 250 V/cm pulses by the electroporation Trigrid device to increase transfection efficiency (ICHOR medical systems, San Diego, CA). Mice were given a prime-boost immunization intramuscularly (IM), spaced two weeks apart. For the protein subunit vaccine group, purified HexaPro proteins (0.5 μg or 5 μg per mouse) were mixed with an equal volume of AD03 (AddaS03™; InvivoGen; San Diego, CA) and immunized via IM. For the mRNA group, mice were vaccinated via IM injection with 0.5 μg or 5 μg of mRNA-1273 (a clinical leftover). Sera were collected for analysis on days 5, 29, and 43 after the initial immunization.

#### SARS-CoV-2 cPass neutralization assay

Neutralization antibody detection was performed using the Genscript cPass neutralization assay kit according to the manufacturer’s protocol.^45^ In brief, samples and supplied controls were diluted 1:10 with dilution buffer and mixed with RBD-HRP with a volume ratio of 1: 1. After a 30 min incubation at 37°C, 100 μl of sample dilutions or controls were added to a 96-well plate pre-coated with recombinant hACE2 protein. The plate was incubated for 15 min at 37°C, the sample mixture was removed, and wells were washed four times with 300 μl wash buffer. After the addition of substrate, the reaction was stopped, and plates immediately read at 450 nm. Data were interpreted as percentage reduction (% neutralization) based on OD_450_ intensity. The manufacturer recommended a cut-off of ≥30% signal reduction was used to indicate the presence of anti-SARS-CoV-2 neutralizing antibodies (nAbs). Each sample’s value (% neutralization) was calculated as 100 x (1-OD450_Sample_)/Average OD450_Negative control_.

#### Flow cytometry

293T cells expressing HexaPro Spike or immunized mouse splenocytes collected were mashed using a cell strainer, added to the plate (3×10^5^/well), and stimulated using the Spike peptide pool (5 μg/ml) for three days. Single cells were then incubated with PMA (50 ng/ml) and ionomycin (1 μg/ml), and cytokine release was prevented by treatment with brefeldin A. After stimulation, cells were stained with Live/Dead violet for viability. Anti-Mouse-CD4 (Biolegend#100515), CD8 (Biolegend#100733), IFN-γ (Biolegend#505807), and TNFα (Biolegend#506315) were used for surface and intracellular staining. All flow cytometry data were obtained in Cytec™ NL-3000 (Cytec Biosciences, Fremont, CA) and analyzed with FlowJo software (FlowJov10.8.1). Data were exported and analyzed in GraphPad Prism version 8 (GraphPad Software, San Diego, CA).

#### Preparation of splenocytes

The spleens were aseptically removed and placed in ice-cold RMPI/FBS medium. To generate a single cell suspension, the organ is placed into a 100 μm cell strainer mesh in a petri dish containing 5 ml ice-cold RPMI/FBS and meshed the organ using the plunger of a 3 mL syringe, and transferred the cell suspension in to 15-ml tube, and centrifuged at 300 g for 5 minutes at 4°C. Then, pellet was re-suspended with ACK (Ammonium-Chloride-Potassium) lysis buffer. The lysis was stopped with 5 ml of ice-cold RPMI/FBS and centrifuged at 300 g for 5 minutes. The splenocytes were cultured in RPMI containing 200 U/mL IL-2.

#### IgG ELISA

For binding and quantifying serum antibodies, ELISA 96-well plates (Corning#9018, USA) were coated with 5 μg/ml of the Spike protein overnight at 4°C and blocked in 5% skim milk in PBS for 2 h at RT. Next, plates were washed and incubated with diluted mouse sera (1:10,000) for 2 h. Following primary serum incubation, plates were washed and incubated with HRP-conjugated anti-mouse IgG (Cell Signaling Technology #7076), IgG1 (Abcam #ab97240), IgG2c (Cell Signaling Technology #56970S), and IgG2b (Abcam #ab97250) and IgA (Invitrogen #626720) antibodies for 1 h at room temperature. The plates were developed with 3, 3′, 5, 5′-tetramethylbenzidine (TMB; Sigma #T0440). The reactions were stopped with 1 N hydrochloric acid, and the absorbance was measured at 450 nm using a microplate reader (CLARIOstar- BMG Labtech).

#### TNF-α/IFN-γ ELISpot assay

ELISpot assays were performed according to the manufacturer’s instructions (CTL-Murine IFN-γ/TNF-α Double-Color Enzymatic ELISPOT Assay). Spleens from immunized mice were harvested and stored in RPMI 1640 media before being dissociated by a stomacher. RBCs were removed by ACK lysis buffer. The splenocytes were filtered and counted. 3x10^5^/mL splenocytes were plated into each well and stimulated for 24 h with 15-mer peptides (overlapping by 11 amino acids) spanning the full-length protein sequence of the SARS-CoV-2 Spike (Stemcell, Catalog #100-0676) at a concentration of 5 μg/ml in the ELISpot plate (precoated with IFNγ and TNFα capture antibodies). As a negative control, cells were not stimulated by the peptides. Cells were washed off, and the plates were developed using a biotinylated anti-TNFα and FITC anti-IFNγ detection antibodies followed by a streptavidin-enzyme conjugate or FITC-HRP at 1:1000 dilution resulting in visible spots. After the plates were developed, spots were scanned and quantified using an ImmunoSpot CTL reader (Shaker Heights, OH). Blue spots represent TNFα spots, and red spots represent IFNγ spots. Spot-forming unit (SFU) per well was calculated, and values are shown as a background-subtracted average of measured samples.

### Quantification and statistical analysis

All statistical analyses were performed using GraphPad Prism 8 software. Data are presented as means ± standard deviation (SD). Comparisons between two groups were analyzed by the Student’s *t* test, whereas that for three or more groups were tested using the one-way analysis of variance. Multiple variable correlations were performed using Pearson’s coefficient correlation in the GraphPad Prism software. A probability value of p ≤ 0.05 was considered statistically significant.

## Data Availability

• All the data supporting the findings of this study are available within the paper and are available from the lead contact upon request. • This paper does not report original code. • Any additional information required to reanalyze the data reported in this paper is available from the lead contact upon request.

## Supplemental information

Document S1. Figures S1–S5

Table S1. Reported DNA vaccines against SARS-CoV-2
