# Supplementary material for: Comparison of DNA vaccines with AddaS03 as an adjuvant and an mRNA vaccine against SARS-CoV-2
Source: iScience. 2023 Jun 16;26(7):107120. doi: 10.1016/j.isci.2023.107120 (PMC10271916; doi:10.1016/j.isci.2023.107120)
Supplement: Document S1. Figures S1–S5 [file mmc1.pdf]

iScience, Volume *26*

Supplemental information

Comparison of DNA vaccines with AddaS03 as an adjuvant and an mRNA vaccine against SARS-CoV-2

Praveen Neeli, Dafei Chai, Xu Wang, Navid Sobhani, George Udeani, and Yong Li

iScience, Volume *26*

Supplemental information

Comparison of DNA vaccines with AddaS03 as an adjuvant and an mRNA vaccine against SARS-CoV-2

Praveen Neeli, Dafei Chai, Xu Wang, Navid Sobhani, George Udeani, and Yong Li

Supplemental Information

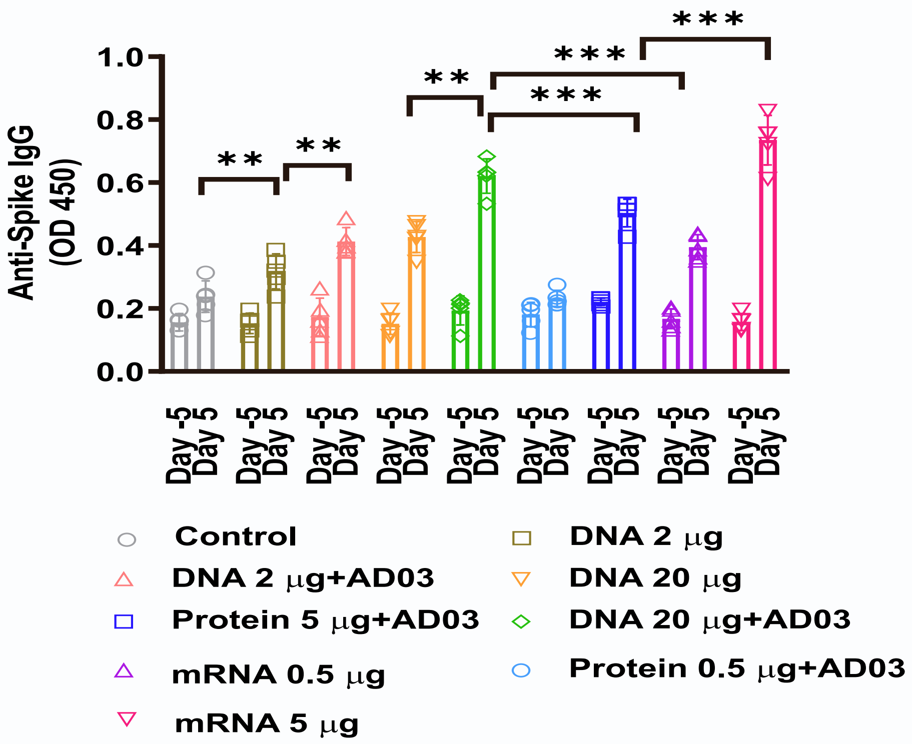

Figure S1. Spike-DNA vaccination induces anti-spike IgG immune response in C57BL/6 mice. Related to Figure 2. Anti-Spike IgG levels were measured in immunized mouse sera before and after 5 days post-first immunization by ELISA (Sera diluted to 1:1,000). Significant differences (**P<0.01; *** P<0.001; two-tailed unpaired t-test) among other groups are shown in the corresponding figures, and the data are presented as means ± SD of at least three samples.

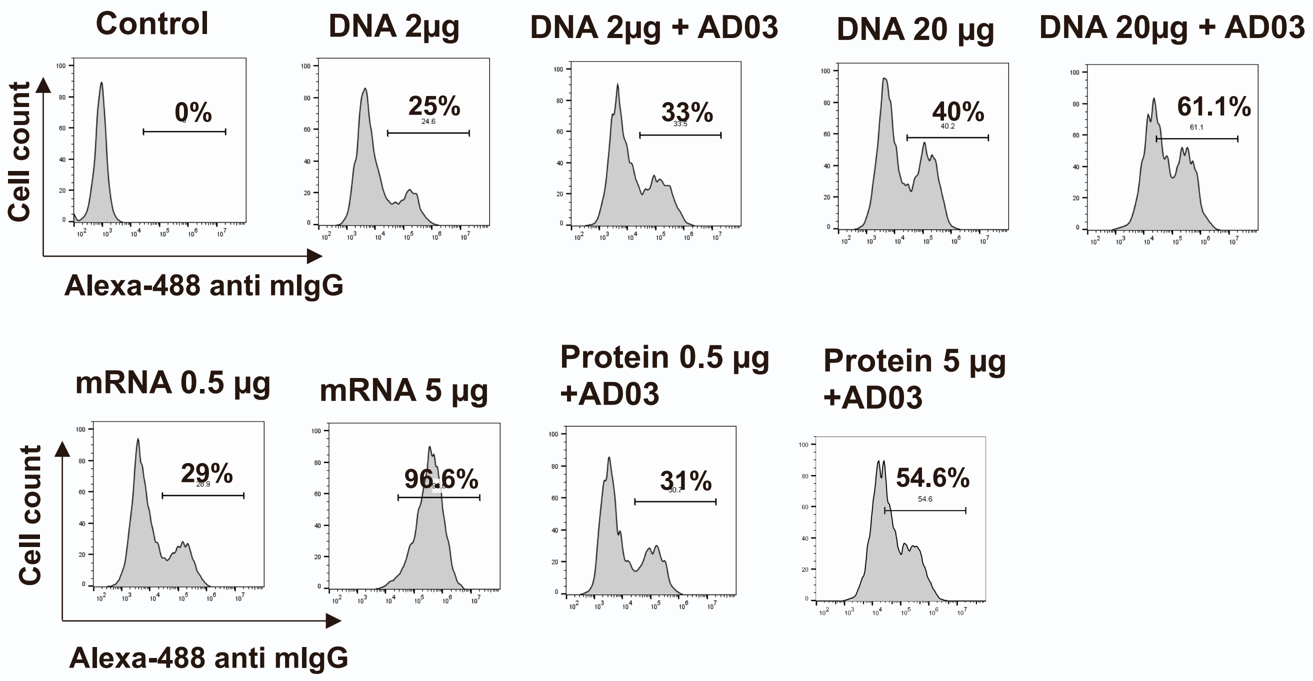

Figure S2. Antibody responses induced by Spike-DNA vaccination in C57BL/6 mice. Related to Figure 2. Flow cytometric analysis of the efficacy of hyper immune mouse sera in binding the Spike antigen expressed on the surface of Spike-expressing 293T cells. 293T cells transfected with Spike-plasmid and later stained with pooled sera of immunized mice. Shown are representative flow cytometry plots to detect the binding efficacy of hyperimmune mouse sera to the HexaPro Spike protein expressed on the surface of 293T cells.

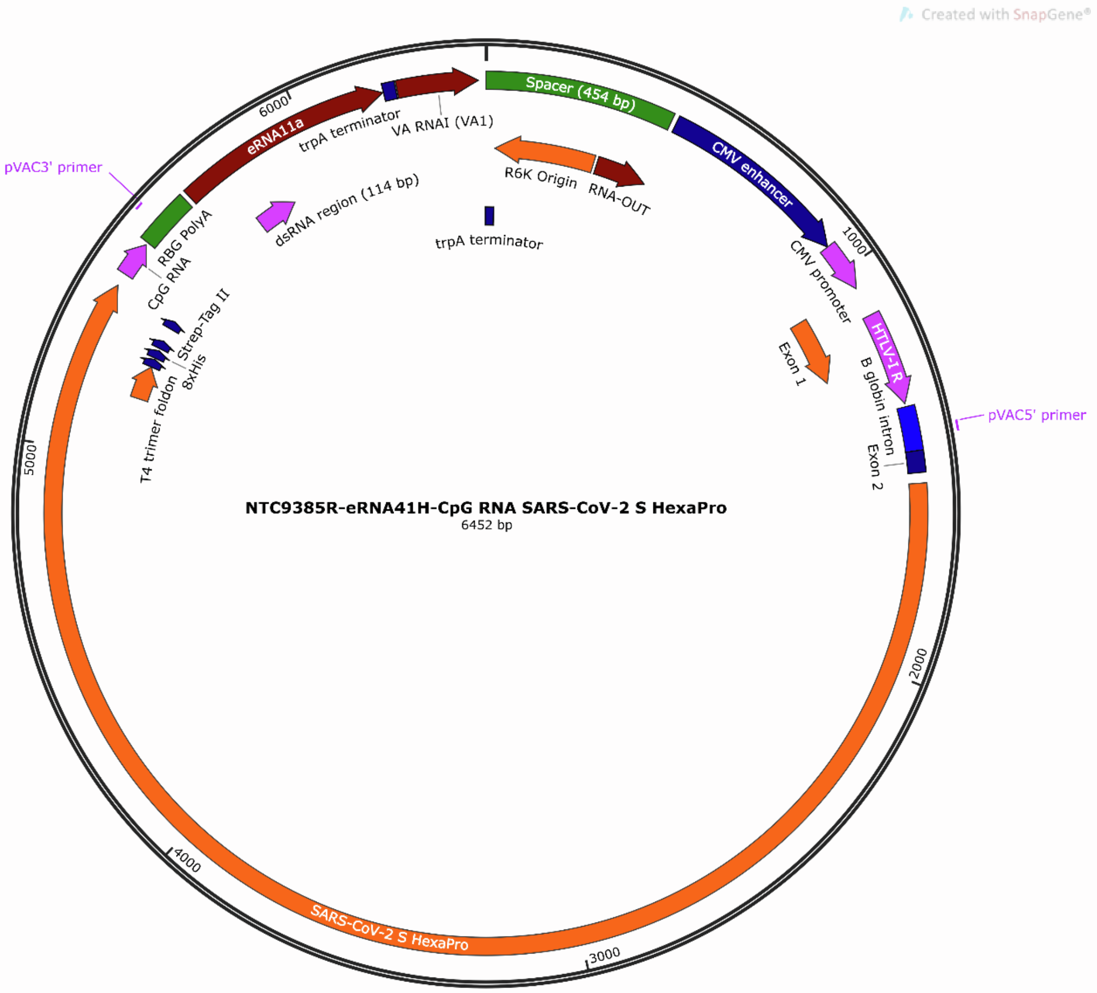

Figure S3. Nano-DNA NTC9385R-SARS-CoV-2 plasmid (6452 bp). **Related to Figure 6**

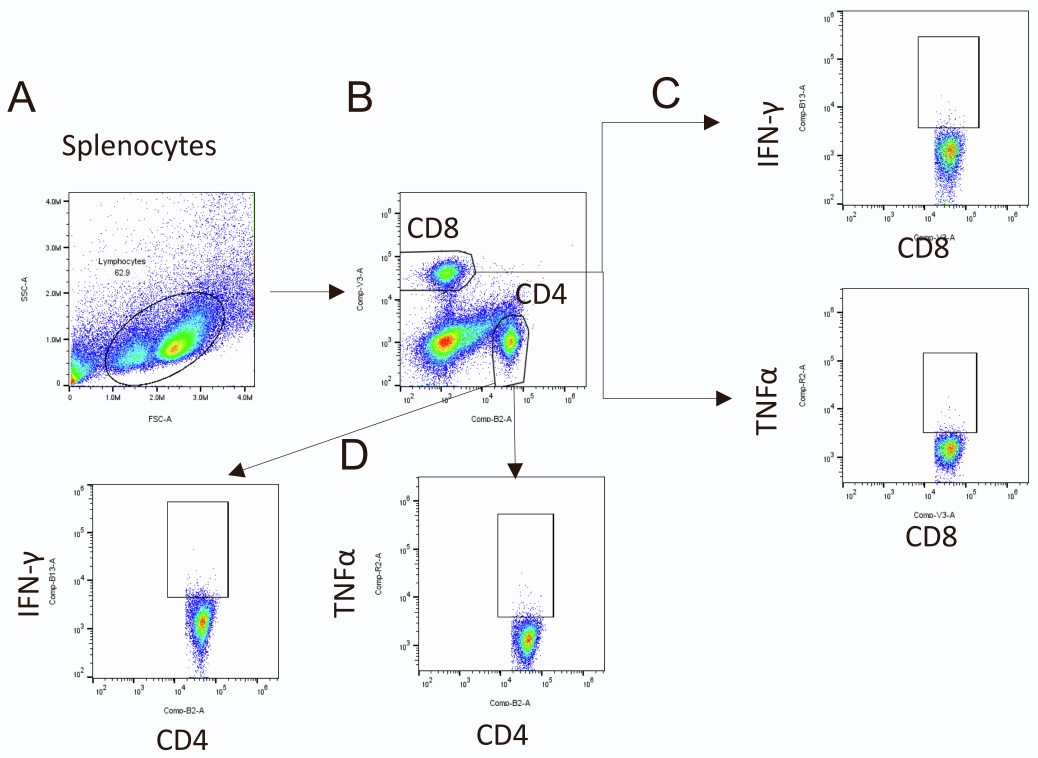

Figure S4. Gating strategy for the analysis of multifunctional T cells. Related to Figure 4. After single cell selection, lymphocytes were gated according to A (FSC-A size) vs. SSA-A (granularity), followed by B to gate CD4+ T cells and CD8+ T cells. (C) CD8+ T cells were plotted against individual cytokine (TNF-α, and IFN-γ). (D) CD4+ T cells were plotted against individual cytokine (TNF-α, and IFN-γ)

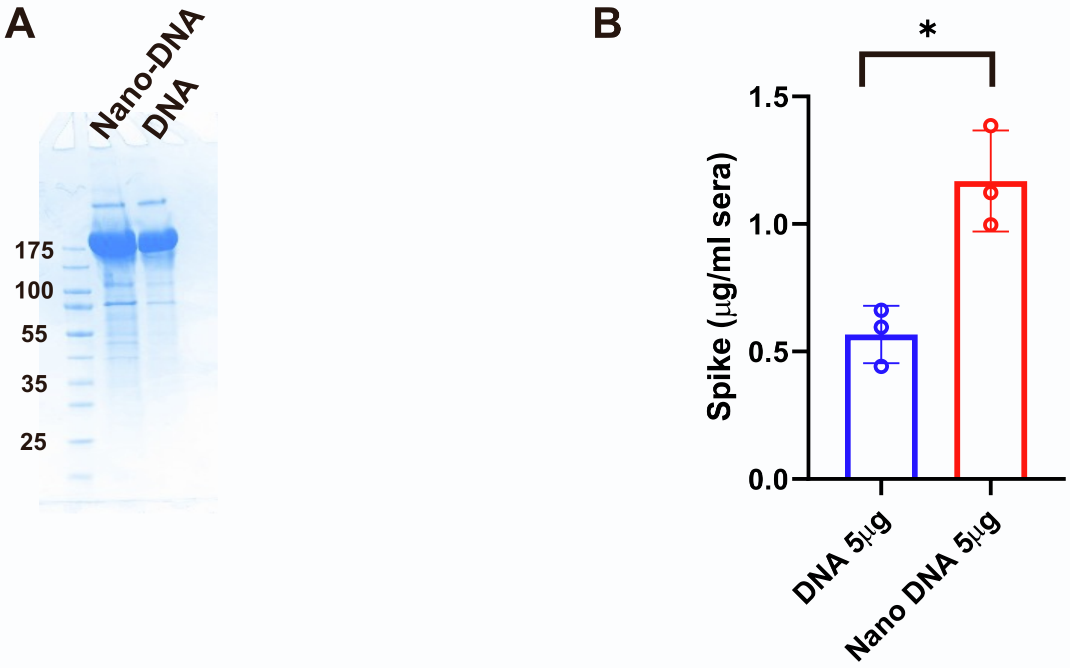

Figure S5. Nano-DNA expression levels are significantly more compared to DNA-Spike plasmid. Related to Figure 6. (**A**) Expi293 transfected with Nano-Spike or DNA-Spike plasmids (1µg/mL); after 5 days post-transfection, Spike- protein was purified and the yield was quantified by SDS-PAGE. Nano-DNA yield was ~2-fold higher than DNA. (**B**) Spike antigen levels in immunized mice sera 5 days post-first vaccination. Significant differences (* $P<0.05$; two-tailed unpaired t-test) are shown in the corresponding figures, and the data are presented as means ± SD of at least three samples.
