# Supplementary material for: Comparison of DNA vaccines with AddaS03 as an adjuvant and an mRNA vaccine against SARS-CoV-2
Source: iScience. 2023 Jun 16;26(7):107120. doi: 10.1016/j.isci.2023.107120 (PMC10271916; doi:10.1016/j.isci.2023.107120)
Supplement: Table S1. Reported DNA vaccines against SARS-CoV-2 [file mmc2.pdf]

Table S1 Reported DNA vaccines against SARS-CoV-2

Antigen [reference]	vector	delivery	antigen dose	adjuvant	nAb	T cell	conclusion
RBD of Spike with Envelope and Nuclear protein [1]	pVAX1	IM-EP	2 times with 50 µg	No. QS21 used in protein	Yes	Yes	Universal vaccine for WH01, Beta, Delta, and Omicron variants
Spike protein [2]	pVAX1	IM-pyro-drive jet injector	2 times with 80 µg	No	Yes (70%)	Yes	Intradermal pVAX1-Spike is a promising vaccine for COVID19.
Spike protein [3]	pVAX1	IM	2 times with 200 µg	Polyinosinic-polycytidylic acid	Yes	Yes	Optimization of spike sequence is important for vaccine design
Spike without C-terminal domain [4]	pCMVkan	IM-EP (DNA-liposome)	3 times with 100 µg	No	Moderate	Yes	Liposome formulation with DNA spike used as a COVID-19 vaccine
Spike of Wuhan strain and omicron strain [5]	pJW4303	IM	2 times with 20 µg of Wuhan Spike, and 50 µg of omicron Spike	No	Yes	Yes	The omicron-matched vaccine is preferred for boosting cross-protective immunity
Full-length Spike or truncated Spike SΔCD, SΔTM, S1 only [6]	pVAX1	IM-EP	3 times with 50 µg plasmid	No	Yes	Yes	Three immunizations with 50 µg antigen were effective against SARS-CoV-2 in mice
Spike and Nucleocapsid [7]	pCAGGS	IM and intranasal (IN) (Quil-A chitosan-	2 times with 50 µg plasmid	No	Yes	Yes	Vaccine efficacy is better in the IM route than IN vaccination

		nanoparticle)					
Combination of inactivated virus vaccines and DNA for Spike RBDs of Wuhan-Hu-1 and Beta, Delta, Lambda strains [8]	pVAX1	ID	3 times with 100 µg plasmid	No	Yes	Yes	The DNA vaccines enhanced the humoral and cellular immune responses of inactivated virus vaccines
Spike [9]	pVAX1	IM-EP	2 times with 100 µg plasmid	No	Yes	Yes	DNA vaccines delivery by Electro acupuncture is effective against SARS-CoV-2
Spike S1+S2 D614G (HexaPro) [10]	pCDNA3.1	IM-EP	3 times with 40 µg plasmid	No	Yes	No	Humoral immunity induced by Spike S1/S2 DNA vaccine
Spike-plasmid and S protein subunit vaccine [11]	pJW4303	IM-injection	3 times with 50 µg plasmid	No (Alum was used for a protein control)	Yes	Yes	Changing the site of IM-injection can significantly improve the immunogenicity of DNA vaccines.
Spike with a immunogenic peptide [12]	pIDV-II	IM-EP	2 times with 50 µg plasmid	5mer4 pentamer-peptide	Yes	Yes	DNA-plasmids showed robust immunogenicity against SARS-CoV-2
Spike [13]	INO-4800	ID-EP	2 times with 2 mg and 1 mg (rhesus macaques)	No	Yes	Yes	INO-4800 and INO-4802 provide heterologous protection against emerging variants in rhesus macaques.
Spike protein fused to Xcl1 [14]	pVAX1	IM-EP	2 times with 25 µg plasmid	Xcl1	Yes	Yes	Xcl1-Spike expressed in cDC1 induced potential antibody and T-cell response.

Envelope and Membrane proteins [15]	pcDNA3.1	IM-EP	3 times with 35 µg plasmid	No	No	Yes	E/M proteins can be a potential vaccine candidate against SARS-CoV-2.
RBD-Spike-DNA vaccine mixed with RBD conjugated to polyglucin: spermidine [16]	pVAX1	IM	2 times (100 µg DNA+ 100 µg RBD conjugate)	No	Yes	No	Combination of DNA vaccine and RBD protein synergistically increases humoral response to RBD protein in mice.
RBD coated with N-linked glycan [17]	pVAX1 (Self-assembled nanoparticle)	IM-EP	1 time with 1 or 5 µg of DNA expressing RBD g5.1 24 or 120-mer	No	Yes	Yes	Single-dose nanovaccines generated potent immune responses against SARS-CoV-2
Spike [18]	pVAX1, pGX9501	IM	2 times with 25 µg plasmid	No	Yes	Yes	pGX-9501 was more protective than pVAX1 vector in protecting the mice against SARS-CoV-2
RBD of Spike [19]	pVAX1	IM	3 times with 100 µg plasmid	No	Yes	moderate	pVAX-RBD-PGS (pVAX-RBD enclosed in the polyglucine-spermidine conjugate envelope) was better than pVAX-RBD
Spike RBD fused to a soluble PD1 domain (PD1-RBD-DNA/LAIV-HK68-RBD)[20]	pVAX1	IM-EP, intranasal.	1 dose of 50 µg PD1-RBD-DNA by EP; for intranasal at	No	Yes	Yes	LAIV-CA4-RBD could boost the BioNTech vaccine for improved mucosal immunity

			10^6 PFU per mouse				
Spike [21]	NTC8685-eRNA41H	ID using PharmaJet ® Tropis ID device	3 times with 10 µg and 50 µg doses respectively	No. Gerbu Adjuvans MM used in protein	Yes	Yes	The DNA vaccine is immunogenic using ID or IM needle-free delivery methods.
Spike [22]	pCAGGS	ID and Micro-needle EP (ePatch)	2 times each with 10 µg and 100 µg DNA	No	Yes	No	The antibody responses from ePatch were strong and enabled a 10-fold dose sparing compared to conventional EP.
Spike [23]	pVAX1	IM by needle or PharmaJet	3 times with 100 µg DNA	No	yes	yes	The vaccine is highly immunogenic and capable of inducing a long-lasting Th1-skewed humoral and cellular response in mice
Spike DNA and/or Spike-RBD Protein co-immunization [24]	pCMV.kan	IM-EP	2 times with 25 µg DNA for mice and 2mg for rhesus macaques	No. TLR-4 agonist (GLA-SE) used in protein	yes	yes	The Spike DNA with Protein co-immunization induced the highest binding and neutralization antibodies production against SARS-CoV-2
Spike RBD (COVID-eVax) [25]	pTK1A-TPA	IM-EP	2 times each with 10 µg DNA (mice) or 400 µg (ferrets)	No	yes	yes	COVID-eVax conferred significant protection to ferrets upon SARS-CoV-2 challenge.
Spike Protein [26]	pSV10	IM-EP	3 times with 50 µg DNA	BC01	yes	yes	DNA Vaccine with BC01 (an adjuvant derived from unmethylated CpG motif-containing DNA fragments from the Bacillus Calmette-

							Guerin genome) induced an early immune response against SARS-CoV-2.
Spike [27]	pCMVkan	Cationic Lipoplexes or EP	3 times with 100 µg DNA	No	yes	yes	Both formulations were efficient against the virus in terms of nAb neutralization and T cell response activation
Spike S1 (1-681 aa) [28]	pVAX1	IM-EP (PharmaJet)	3 times with 25 µg or 50 µg DNA	No	yes	yes	Immunizing with three doses of 50 µg pVAX-S1 could elicit a significant immune response.
Spike (ZyCov-D) [29]	pVAX-1	ID using PharmaJet	3 dose regimen with 25 µg plasmid each time (mice); 100 µg (Pig); 500 µg (rabbit)	No	Yes	Yes	The candidate DNA vaccine induced neutralizing antibodies and Th-1 immune response against SARS-CoV-2.
Full length or truncated Spike [30]	VR1012	ID (tail)	3 times with 50 µg DNA or without	No. Alum and Advax-2 used in inactivated virus vaccines	yes	yes	SARS-CoV-2 PsIV with alum promoted Th2-type response, while SARS-CoV-2 PsIV with the Advax-2 promoted Th1-type response.
Spike [31]	pIDV-II	ID-EP	1 dose of 46 µg spike DNA	murine GM-CSF	Yes	Yes	Murine GM-CSF adjuvanted DNA vaccine showed enhanced cellular and humoral immune response against the virus.

Spike of SARS-CoV1 (pSARS-S) and SARS-CoV-2 (pSARSS2-S) [32]	pVAX1	IM-EP by a BTX EP machine	2 times with 100 µg of vector	No	yes	yes	Immunization with pSARS-S and pSARS2-S induced similar levels of antibodies against S2 of SARS-CoV-2. pSARS2-S induced antibodies against RBD of the SARS-CoV-2 and very high nAb titers against SARS-CoV-2
glycosylated RBD (gRBD) of Spike [33]	pCMV/R	IM-EP	2 times with 60 µg of vector	No	yes	yes	gRBD (protein or DNA) had higher immunogenicity than wild-type RBD; multivalent gRBD fusion proteins are more efficient vaccines in a cost-effective way.
Spike protein (S) or SΔTM [34]	pGX27	IM-EP	3 times with 5, 15, and 45 µg (mice) or 3 mg (cynomolgus macaques)	No	yes	yes	The vaccine with Spike without the transmembrane domain provides better immunity against the virus than S.
Spike proteins or S1/S2 [35]	pCMVkan	IM-EP	3 times with 100 µg plasmid	No	yes	yes	Full-length S antigen was more potent than S1 or S2 proteins.
W4P-RBD and RBD only of Spike [36]	pcDNA3.3	IM	3 times with 50 µg plasmid	No	yes	yes	W4P-RBD led to an increased immune response against the virus compared to RBD alone.
Spike (S) [37]	pSW3891	IM by Helio gene gun	3 times with 5 µg (mice), 200 µg (rabbits), and 2 mg	No. Alum used for protein	yes	yes	The co-delivery of DNA and protein components at the same time was equally potent as sequential immunization; and elicited full protection against the virus.

			(rhesus monkeys)				
DNA-launched self-replicating vaccine encoding Spike (DREP-S) or Pre-fusion ectodomain (DREP-S^{ecto}) [38]	Semliki Forest virus	ID-EP	2 times with 10 µg DNA	No. Addavax used in protein	yes	yes	DREP constructs were able to efficiently generate immune responses against the virus.
Spike (S) (INO-4800) [39]	pVAX1	IM-EP	3 dose regiment each dose with 2.5, 10, and 25 µg DNA (mice) or 100 µg (pigs)	No	yes	yes	The vaccine produced a robust expression of the S protein in vitro and robust immune response in immunized mice and guinea pigs.

IM, intramuscular; ID, intradermal; EP, electroporation.

References

1. Appelberg, S., Ahlén, G., Yan, J., Nikouyan, N., Weber, S., Larsson, O., Höglund, U., Aleman, S., Weber, F., Perlhamre, E., et al. (2022). A universal SARS-CoV DNA vaccine inducing highly cross-reactive neutralizing antibodies and T cells. EMBO Mol Med, e15821. 10.15252/emmm.202215821.
2. Nishikawa, T., Chang, C.Y., Tai, J.A., Hayashi, H., Sun, J., Torii, S., Ono, C., Matsuura, Y., Ide, R., Mineno, J., et al. (2022). Immune response induced in rodents by anti-CoVid19 plasmid DNA vaccine via pyro-drive jet injector inoculation. Immunological Medicine, 1-14. 10.1080/25785826.2022.2111905.
3. Li, Z.X., Feng, S., Zhang, H., Zhuang, X.Y., Shang, C., Sun, S.Y., Han, J.C., Xie, Y.B., Zhang, J.Y., Wang, W., et al. (2022). Immunogenicity and protective efficacy of a DNA vaccine inducing optimal expression of the SARS-CoV-2 S gene in hACE2 mice. Arch Virol, 1-10. 10.1007/s00705-022-05562-z.
4. Peletta, A., Prompetchara, E., Tharakhet, K., Kaewpang, P., Buranapraditkun, S., Yostrerat, N., Manopwisedjaroen, S., Thitithanyanont, A., Avaro, J., Krupnik, L., et al. (2022). Translating a Thin-Film Rehydration Method to Microfluidics for the Preparation of a SARS-CoV-2 DNA Vaccine: When Manufacturing Method Matters. Pharmaceutics *14*, 1427.
5. Jia, L., Zhou, Y., Li, S., Zhang, Y., Yan, D., Wang, W., Zhang, W., Wan, Y., and Qiu, C. (2022). Omicron Booster in Ancestral Strain Vaccinated Mice Augments Protective Immunities Against Both Delta and Omicron Variants. Frontiers in Immunology *13*. 10.3389/fimmu.2022.897879.
6. Lim, H., Kim, S.E., Lee, Y.H., Hwang, Y.-H., Kim, S.H., Kim, M.Y., Chung, G.T., Kim, Y.-J., Kim, D., and Lee, J.-A. (2022). Immunogenicity of candidate SARS-CoV-2 DNA vaccines based on the spike protein. Virology *573*, 118-123. https://doi.org/10.1016/j.virol.2022.06.006.
7. Chandrasekar, S.S., Phanse, Y., Riel, M., Hildebrand, R.E., Hanafy, M., Osorio, J.E., Abdelgayed, S.S., and Talaat, A.M. (2022). Systemic Neutralizing Antibodies and Local Immune Responses Are Critical for the Control of SARS-CoV-2. Viruses *14*, 1262.
8. Meng, Z., Ma, D., Duan, S., Zhang, J., Yue, R., Li, X., Gao, Y., Li, X., Zeng, F., Xu, X., et al. (2022). Immunological Study of Combined Administration of SARS-CoV-2 DNA Vaccine and Inactivated Vaccine. Vaccines *10*, 929.
9. Tzeng, T.-T., Chai, K.M., Shen, K.-Y., Yu, C.-Y., Yang, S.-J., Huang, W.-C., Liao, H.-C., Chiu, F.-F., Dou, H.-Y., Liao, C.-L., et al. (2022). A DNA vaccine candidate delivered by an electroacupuncture machine provides protective immunity against SARS-CoV-2 infection. npj Vaccines *7*, 60. 10.1038/s41541-022-00482-0.
10. Wang, X., Rcheulishvili, N., Cai, J., Liu, C., Xie, F., Hu, X., Yang, N., Hou, M., Papukashvili, D., He, Y., and Wang, P.G. (2022). Development of DNA Vaccine Candidate against SARS-CoV-2. Viruses *14*, 1049.
11. Tian, X., Zhang, Y., He, Z., Li, S., Yan, D., Zhu, Z., Wan, Y., and Wang, W. (2022). Successive Site Translocating Inoculation Improved T Cell Responses Elicited by a DNA Vaccine Encoding SARS-CoV-2 S Protein. Front Immunol *13*, 875236. 10.3389/fimmu.2022.875236.
12. Babuadze, G.G., Fausther-Bovendo, H., deLaVega, M.A., Lillie, B., Naghibosadat, M., Shahhosseini, N., Joyce, M.A., Saffran, H.A., Lorne Tyrrell, D., Falzarano, D., et al. (2022). Two DNA vaccines protect against severe disease and pathology due to SARS-CoV-2 in Syrian hamsters. NPJ Vaccines *7*, 49. 10.1038/s41541-022-00461-5.

13. Walters, J.N., Schouest, B., Patel, A., Reuschel, E.L., Schultheis, K., Parzych, E., Maricic, I., Gary, E.N., Purwar, M., Andrade, V.M., et al. (2022). Prime-boost vaccination regimens with INO-4800 and INO-4802 augment and broaden immune responses against SARS-CoV-2 in nonhuman primates. Vaccine *40*, 2960-2969. https://doi.org/10.1016/j.vaccine.2022.03.060.
14. Qi, H., Sun, Z., Yao, Y., Chen, L., and Su, X. (2022). Immunogenicity of the Xcl1-SARS-CoV-2 Spike Fusion DNA Vaccine for COVID-19. Vaccines *10*, 407.
15. Chen, J., Deng, Y., Huang, B., Han, D., Wang, W., Huang, M., Zhai, C., Zhao, Z., Yang, R., Zhao, Y., et al. (2022). DNA Vaccines Expressing the Envelope and Membrane Proteins Provide Partial Protection Against SARS-CoV-2 in Mice. Front Immunol *13*, 827605. 10.3389/fimmu.2022.827605.
16. Borgoyakova, M.B., Karpenko, L.I., Rudometov, A.P., Volosnikova, E.A., Merkuleva, I.A., Starostina, E.V., Zadorozhny, A.M., Isaeva, A.A., Nesmeyanova, V.S., Shanshin, D.V., et al. (2022). Self-Assembled Particles Combining SARS-CoV-2 RBD Protein and RBD DNA Vaccine Induce Synergistic Enhancement of the Humoral Response in Mice. International Journal of Molecular Sciences *23*, 2188.
17. Konrath, K.M., Liaw, K., Wu, Y., Zhu, X., Walker, S.N., Xu, Z., Schultheis, K., Chokkalingam, N., Chawla, H., Du, J., et al. (2022). Nucleic acid delivery of immune-focused SARS-CoV-2 nanoparticles drives rapid and potent immunogenicity capable of single-dose protection. Cell Rep *38*, 110318. 10.1016/j.celrep.2022.110318.
18. Jiang, S., Wu, S., Zhao, G., He, Y., Bao, L., Liu, J., Qin, C., Hou, J., Ding, Y., Cheng, A., et al. (2022). Comparison of Wild Type DNA Sequence of Spike Protein from SARS-CoV-2 with Optimized Sequence on The Induction of Protective Responses Against SARS-Cov-2 Challenge in Mouse Model. Human Vaccines & Immunotherapeutics *18,* 2016201. 10.1080/21645515.2021.2016201.
19. Borgoyakova, M.B., Karpenko, L.I., Rudometov, A.P., Shanshin, D.V., Isaeva, A.A., Nesmeyanova, V.S., Volkova, N.V., Belenkaya, S.V., Murashkin, D.E., Shcherbakov, D.N., et al. (2021). Immunogenic Properties of the DNA Construct Encoding the Receptor-Binding Domain of the SARS-CoV-2 Spike Protein. Mol Biol *55*, 889-898. 10.1134/s0026893321050046.
20. Zhou, R., Wang, P., Wong, Y.C., Xu, H., Lau, S.Y., Liu, L., Mok, B.W., Peng, Q., Liu, N., Woo, K.F., et al. (2022). Nasal prevention of SARS-CoV-2 infection by intranasal influenza-based boost vaccination in mouse models. EBioMedicine *75*, 103762. 10.1016/j.ebiom.2021.103762.
21. Lassaunière, R., Polacek, C., Gram, G.J., Frische, A., Tingstedt, J.L., Krüger, M., Dorner, B.G., Cook, A., Brown, R., Orekov, T., et al. (2021). Preclinical evaluation of a candidate naked plasmid DNA vaccine against SARS-CoV-2. npj Vaccines *6*, 156. 10.1038/s41541-021-00419-z.
22. Xia, D., Jin, R., Byagathvalli, G., Yu, H., Ye, L., Lu, C.-Y., Bhamla, M.S., Yang, C., and Prausnitz, M.R. (2021). An ultra-low-cost electroporator with microneedle electrodes (ePatch) for SARS-CoV-2 vaccination. Proceedings of the National Academy of Sciences *118*, e2110817118. doi:10.1073/pnas.2110817118.
23. Alamri, S.S., Alluhaybi, K.A., Alhabbab, R.Y., Basabrain, M., Algaissi, A., Almahboub, S., Alfaleh, M.A., Abujamel, T.S., Abdulaal, W.H., ElAssouli, M.Z., et al. (2021). Synthetic SARS-CoV-2 Spike-Based DNA Vaccine Elicits Robust and Long-Lasting Th1 Humoral and Cellular Immunity in Mice. Front Microbiol *12*, 727455. 10.3389/fmicb.2021.727455.
24. Rosati, M., Agarwal, M., Hu, X., Devasundaram, S., Stellas, D., Chowdhury, B., Bear, J., Burns, R., Donohue, D., Pessaint, L., et al. (2021). Control of SARS-CoV-2 infection after Spike DNA or Spike DNA+Protein co-immunization in rhesus macaques. PLOS Pathogens *17,* e1009701. 10.1371/journal.ppat.1009701.

25. Conforti, A., Marra, E., Palombo, F., Roscilli, G., Ravà, M., Fumagalli, V., Muzi, A., Maffei, M., Luberto, L., Lione, L., et al. (2022). COVID-eVax, an electroporated DNA vaccine candidate encoding the SARS-CoV-2 RBD, elicits protective responses in animal models. Molecular Therapy *30*, 311-326. 10.1016/j.ymthe.2021.09.011.
26. Zhou, Z., Zhang, X., Li, Q., Fu, L., Wang, M., Liu, S., Wu, J., Nie, J., Zhang, L., Zhao, C., et al. (2021). Unmethylated CpG motif-containing genomic DNA fragments of bacillus calmette-guerin improves immune response towards a DNA vaccine for COVID-19. Vaccine *39*, 6050-6056. 10.1016/j.vaccine.2021.08.103.
27. Peletta, A., Prompetchara, E., Tharakhet, K., Kaewpang, P., Buranapraditkun, S., Techawiwattanaboon, T., Jbilou, T., Krangvichian, P., Sirivichayakul, S., Manopwisedjaroen, S., et al. (2021). DNA Vaccine Administered by Cationic Lipoplexes or by In Vivo Electroporation Induces Comparable Antibody Responses against SARS-CoV-2 in Mice. Vaccines (Basel) *9*. 10.3390/vaccines9080874.
28. Alluhaybi, K.A., Alharbi, R.H., Alhabbab, R.Y., Aljehani, N.D., Alamri, S.S., Basabrain, M., Alharbi, R., Abdulaal, W.H., Alfaleh, M.A., Tamming, L., et al. (2021). Cellular and Humoral Immunogenicity of a Candidate DNA Vaccine Expressing SARS-CoV-2 Spike Subunit 1. Vaccines (Basel) *9*. 10.3390/vaccines9080852.
29. Dey, A., Chozhavel Rajanathan, T.M., Chandra, H., Pericherla, H.P.R., Kumar, S., Choonia, H.S., Bajpai, M., Singh, A.K., Sinha, A., Saini, G., et al. (2021). Immunogenic potential of DNA vaccine candidate, ZyCoV-D against SARS-CoV-2 in animal models. Vaccine *39*, 4108-4116. 10.1016/j.vaccine.2021.05.098.
30. Sundaram, A.K., Ewing, D., Liang, Z., Jani, V., Cheng, Y., Sun, P., Raviprakash, K., Wu, S.-J., Petrovsky, N., Defang, G., et al. (2021). Immunogenicity of Adjuvanted Psoralen-Inactivated SARS-CoV-2 Vaccines and SARS-CoV-2 Spike Protein DNA Vaccines in BALB/c Mice. Pathogens *10*, 626.
31. Vernet, R., Charrier, E., Cosset, E., Fièvre, S., Tomasello, U., Grogg, J., and Mach, N. (2021). Local Sustained GM-CSF Delivery by Genetically Engineered Encapsulated Cells Enhanced Both Cellular and Humoral SARS-CoV-2 Spike-Specific Immune Response in an Experimental Murine Spike DNA Vaccination Model. Vaccines (Basel) *9*. 10.3390/vaccines9050484.
32. Chai, K.M., Tzeng, T.T., Shen, K.Y., Liao, H.C., Lin, J.J., Chen, M.Y., Yu, G.Y., Dou, H.Y., Liao, C.L., Chen, H.W., and Liu, S.J. (2021). DNA vaccination induced protective immunity against SARS CoV-2 infection in hamsterss. PLoS Negl Trop Dis *15*, e0009374. 10.1371/journal.pntd.0009374.
33. Guo, Y., He, W., Mou, H., Zhang, L., Chang, J., Peng, S., Ojha, A., Tavora, R., Parcells, M.S., Luo, G., et al. (2021). An Engineered Receptor-Binding Domain Improves the Immunogenicity of Multivalent SARS-CoV-2 Vaccines. mBio *12*. 10.1128/mBio.00930-21.
34. Seo, Y.B., Suh, Y.S., Ryu, J.I., Jang, H., Oh, H., Koo, B.S., Seo, S.H., Hong, J.J., Song, M., Kim, S.J., and Sung, Y.C. (2021). Soluble Spike DNA Vaccine Provides Long-Term Protective Immunity against SARS-CoV-2 in Mice and Nonhuman Primates. Vaccines (Basel) *9*. 10.3390/vaccines9040307.
35. Prompetchara, E., Ketloy, C., Tharakhet, K., Kaewpang, P., Buranapraditkun, S., Techawiwattanaboon, T., Sathean-Anan-Kun, S., Pitakpolrat, P., Watcharaplueksadee, S., Phumiamorn, S., et al. (2021). DNA vaccine candidate encoding SARS-CoV-2 spike proteins elicited potent humoral and Th1 cell-mediated immune responses in mice. PLoS One *16*, e0248007. 10.1371/journal.pone.0248007.
36. Jeong, H., Choi, Y.M., Seo, H., and Kim, B.J. (2021). A Novel DNA Vaccine Against SARS-CoV-2 Encoding a Chimeric Protein of Its Receptor-Binding Domain (RBD) Fused to the Amino-Terminal Region of Hepatitis B Virus preS1 With a W4P Mutation. Front Immunol *12*, 637654. 10.3389/fimmu.2021.637654.

37. Li, Y., Bi, Y., Xiao, H., Yao, Y., Liu, X., Hu, Z., Duan, J., Yang, Y., Li, Z., Li, Y., et al. (2021). A novel DNA and protein combination COVID-19 vaccine formulation provides full protection against SARS-CoV-2 in rhesus macaques. Emerg Microbes Infect *10*, 342-355. 10.1080/22221751.2021.1887767.
38. Szurgot, I., Hanke, L., Sheward, D.J., Vidakovics, L.P., Murrell, B., McInerney, G.M., and Liljeström, P. (2021). DNA-launched RNA replicon vaccines induce potent anti-SARS-CoV-2 immune responses in mice. Sci Rep *11*, 3125. 10.1038/s41598-021-82498-5.
39. Smith, T.R.F., Patel, A., Ramos, S., Elwood, D., Zhu, X., Yan, J., Gary, E.N., Walker, S.N., Schultheis, K., Purwar, M., et al. (2020). Immunogenicity of a DNA vaccine candidate for COVID-19. Nat Commun *11*, 2601. 10.1038/s41467-020-16505-0.
